# Analysis on multi-domain cooperation for predicting protein-protein interactions

**DOI:** 10.1186/1471-2105-8-391

**Published:** 2007-10-16

**Authors:** Rui-Sheng Wang, Yong Wang, Ling-Yun Wu, Xiang-Sun Zhang, Luonan Chen

**Affiliations:** 1 School of Information, Renmin University of China, Beijing 100872, China; 2 Department of Electronics Information and Communication Engineering, Osaka Sangyo University, Osaka 574-8530, Japan; 3 Academy of Mathematics and Systems Science, Chinese Academy of Sciences, Beijing 100080, China; 4 Institute of Systems Biology, Shanghai University, Shanghai 200444, China; 5 ERATO Aihara Complexity Modelling Project, JST, Tokyo 151-0064, Japan; 6 Institute of Industrial Science, The University of Tokyo, Tokyo 153-8505, Japan

## Abstract

**Background:**

Domains are the basic functional units of proteins. It is believed that protein-protein interactions are realized through domain interactions. Revealing multi-domain cooperation can provide deep insights into the essential mechanism of protein-protein interactions at the domain level and be further exploited to improve the accuracy of protein interaction prediction.

**Results:**

In this paper, we aim to identify cooperative domains for protein interactions by extending two-domain interactions to multi-domain interactions. Based on the high-throughput experimental data from multiple organisms with different reliabilities, the interactions of domains were inferred by a Linear Programming algorithm with Multi-domain pairs (LPM) and an Association Probabilistic Method with Multi-domain pairs (APMM). Experimental results demonstrate that our approach not only can find cooperative domains effectively but also has a higher accuracy for predicting protein interaction than the existing methods. Cooperative domains, including strongly cooperative domains and superdomains, were detected from major interaction databases MIPS and DIP, and many of them were verified by physical interactions from the crystal structures of protein complexes in PDB which provide intuitive evidences for such cooperation. Comparison experiments in terms of protein/domain interaction prediction justified the benefit of considering multi-domain cooperation.

**Conclusion:**

From the computational viewpoint, this paper gives a general framework to predict protein interactions in a more accurate manner by considering the information of both multi-domains and multiple organisms, which can also be applied to identify cooperative domains, to reconstruct large complexes and further to annotate functions of domains. Supplementary information and software are provided in  and .

## Background

Many proteins involved in signal transduction, gene regulation and other biological activities require interaction with other proteins or cofactors to achieve specific processes [1,2]. Elucidating protein-protein interactions can provide deep insights into protein functions and intracellular signaling pathways. Owing to the recent rapid advances in high-throughput technologies, protein-protein interaction data of various species are increasingly accumulated from different experiments and deposited in several main databases such as DIP [3] and MIPS [4]. This collection of protein-protein interaction data results in a rich, but quite noisy and still incomplete source of information [5,6] which needs to be analyzed and completed by sophisticated computational methods.

In recent years, a number of computational algorithms have been developed to infer protein-protein interactions, such as those methods based on gene fusion (Rosetta Stone) [7,8], phylogenetic profile [9], protein structure [10], and domain information [11]. In particular, inferring protein-protein interactions (PPI) based on domain information, such as association method [11], probabilistic method [12-14], SVM-based method [15], and LP-based approach [16], has attracted much attention due to its clear biological implication and simplicity. In addition to these methods for protein interaction prediction, inferring domain-domain interactions (DDI) by integrating multiple data sources has also been investigated [17-19].

Domain-based protein interaction prediction assumes that proteins are composed by a set of recognition elements which are referred to as domains, and protein-protein interactions are achieved through domain interactions [12]. A typical procedure for these methods includes two steps. Firstly domain interactions are inferred from experimental protein interactions, and then new protein interactions are predicted based on the inferred domain interactions according to either a probabilistic or deterministic model. The difference between probabilistic and deterministic models is whether or not they are based on the probabilistic formula describing the relations between domain interactions and protein interactions [12]. Most existing algorithms consider domain-domain pairs as the basic units of protein-protein interactions, and these domain-domain interactions are assumed to be independent. However, such an assumption is actually not biologically reasonable because two or more domains may cooperatively interact with another domain [20]. In addition, there are many superdomains where two domains always appear together in individual proteins to mediate the interactions. Given the close relations between two domains in a superdomain, the independence assumption of domain-domain interactions does not generally hold. For example, domain 4 of RNA polymerase Rpb1 (PF05000) and domain 1 of RNA polymerase Rpb1 (PF00623) constitute a superdomain, and they always appear together in individual proteins such as YOR341W, YDL140C and YOR116C, and have many common domain interaction partners [21].

Recently, Han et al. studied domain combinations in protein interactions [22,23]. In their work, the appearance frequencies of domain combinations in a set of interacting and non-interacting protein pairs are counted to construct AP (Appearance Probability) matrices [22,23] which provide useful information about the distribution of multi-domain interactions. For example, among the listed 300 domain combination pairs with high appearance probability values (top300) which are counted based on the total 5826 protein interaction pairs in yeast, there are 246 two-domain pairs, 44 three-domain pairs and 10 four and above domain pairs. Such statistical result indicates that many domains are closely correlated and tend to appear in interacting protein pairs together. In addition, Wang and Caetano-Anolles [24] used the occurrence and abundance of the molecular interactome of domain combinations to construct global phylogenic trees. When a closely correlated domain combination appears in an interactome, domains in this combination may mediate the interaction simultaneously and cooperatively.

Similar to proteins in a complex which cooperatively bind to each other so as to achieve specific functions [1], there is also such a cooperation among domains in protein interactions. For example, Klemm and Pabo [25] found that two unlinked polypeptides corresponding to the POU-specific domain and the POU homeo domain in protein Oct-1 bind cooperatively to the octamer site. Moza et al. [20] showed that the binding energetics between different hot regions consisting of interfacial residues in a protein-protein interaction are not strictly additive. Cooperative binding energetics between distinct hot regions is significant. They pointed out that cooperativity between hot regions has significant implications for the prediction of protein-protein interactions. When the hot regions are distributed over different domains in proteins, the cooperativity between different hot regions is actually embodied by multi-domain cooperation. Hence, revealing such domain cooperation may provide deep insights into the essential mechanism of protein interactions at the domain level, and can also be further exploited to improve the accuracy of protein interaction prediction.

In this paper, we firstly aim to identify cooperative domains from protein interaction data by extending two-domain interactions to multi-domain interactions. Cooperative domains mean that the strength of their cooperative interaction with some domain is stronger than the corresponding domain-domain interactions. Then, by employing the information of both multi-domains and multiple organisms, we propose a general framework based on a Linear Programming with Multi-domain pairs (LPM) and an Association Probabilistic Method with Multi-domain pairs (APMM), to predict protein interactions in a more accurate manner. Experimental results demonstrate that our approach not only can identify cooperative domains effectively but also has a higher accuracy for predicting protein interactions than the existing methods. Cooperative domains, including strongly cooperative domains and superdomains, were detected from major interaction databases, e.g. MIPS and DIP, and many of them were verified by checking physical interactions from the crystal structures of protein complexes in PDB (Protein Data Bank). These crystal structures of complexes provide intuitive evidences for such cooperation. In addition, comparison experiments in terms of protein/domain interaction prediction also justified the benefit of considering multi-domain cooperation.

## Results

**Figure 1:**
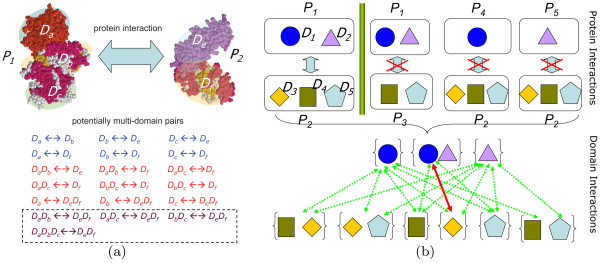
An illustrative example for multi-domain interactions. (a) All multi-domain pairs are listed for two proteins *P*_1 _and *P*_2_. Proteins: *P*_1 _= {*D*_*a*_, *D*_*b*_, *D*_*c *_}, *P*2 = {*D*_*e*_, *D*_*f*_} ; Domains: *D*_*a*_, *D*_*b*_, *D*_*c*_, *D*_*e*_, *D*_*f*_; (b) The illustration of domain interactions by considering multi-domain pairs in the proposed model. There are one pair of interacting proteins and three pairs of non-interacting proteins. The bold line (red) represents interacting domain pair, while the dotted lines (green) are the deleted non-interacting domain pairs.

In this paper, we investigate domain cooperation in protein interactions by extending two-domain interactions to multi-domain interactions. We first define the types of domains and domain pairs, and then describe the main results. Assume that there are *M *domains *D*_1_, …, *D*_*M *_involved in the experimental interaction data. We use (*D*_*m*_, *D*_*n*_) to represent a two-domain pair, one domain in a protein and the other in another protein, and use (*D*_*m*_*-D*_*r*_, *D*_*n*_) to denote a generalized pair i.e. a three-domain pair, where *D*_*m *_and *D*_*r *_appear in one protein (denoted by *D*_*m*_*-D*_*r*_) and *D*_*n *_in another protein. A multi-domain pair means a two-domain pair or a three-domain pair. In Figure 1(a), we list all the multi-domain pairs in proteins (*P*_1_, *P*_2_). A cooperative-domain pair (cooperative-domain interaction) implies a generalized pair (*D*_*m*_*-D*_*r*_, *D*_*n*_) in which two domains *D*_*m*_*-D*_*r *_referred as cooperative domains coexist in a protein *P*_1 _and cooperatively interact with *D*_*n *_in another protein *P*_2_. The cooperative-domain pair should have a stronger interaction effect than the corresponding two-domain pairs (*D*_*m*_, *D*_*n*_) and (*D*_*r*_, *D*_*n*_). A strongly cooperative-domain pair (strongly cooperative-domain interaction) is a cooperative-domain pair (*D*_*m*_*-D*_*r*_, *D*_*n*_) which satisfies that there is an interaction effect of *D*_*m *_or *D*_*r *_on *D*_*n *_only if the domains *D*_*m *_and *D*_*r *_appear together. In Figure 1(b), *D*_1 _and *D*_2 _are strongly cooperative domains interacting with *D*_3 _because all other domain pairs involving *D*_1 _or *D*_2 _have no interactions. A superdomain implies two 'combined' domains *D*_*m*_*-D*_*r *_which are special cooperative domains and always appear together in individual proteins. Note that we extend two-domain interactions only to three-domain interactions because the cooperation of more than three domains is believed to be rare compared with the cases of two and three domains according to statistics [22].

The concept of cooperative domains in our work seems to be similar to Han et al.'s "domain combination" [22,23]. However, there are two fundamental differences between these two concepts. Firstly, the definition of domain combination is not related to domain interaction strength. Each domain combination pair is considered in their approach, no matter what its appearance frequency is in interacting and non-interacting protein pairs. In contrast, the definition of cooperative domains emphasizes "cooperation" and is related to domain interaction strength. By an elaborated variable selection strategy (see Methods), only when the interaction strength of a three-domain pair (*D*_*m*_*-D*_*r*_, *D*_*n*_) is larger than those of the corresponding two-domain pairs (*D*_*m*_, *D*_*n*_) and (*D*_*r*_, *D*_*n*_), this three-domain pair can possibly be a cooperative-domain pair and considered in the model. Secondly, there is no redundant correlation between different domain combinations in our work. For example, if a three-domain pair (*D*_*m*_*-D*_*r*_, *D*_*n*_) is considered in our method according to the rules of selecting variables (i.e. *D*_*m*_, *D*_*r *_are considered as cooperative domains), the two-domain pairs (*D*_*m*_, *D*_*n*_) and (*D*_*r*_, *D*_*n*_) will not be included into the consideration as interacting domains in the same protein pair to eliminate the redundancy, in contrast to the high correlation among Han et al.'s domain combinations [22,23].

In the following text, Pr(*d*_*m*, *n *_= 1) represents the probability that domain *D*_*m *_interacts with *D*_*n*_. Pr(*d*_*mr*, *n *_= 1) represents the probability that domains *D*_*m *_and *D*_*r *_cooperatively interact with *D*_*n*_. Similarly, Pr(*d*_*m*, *nr *_= 1) represents the probability that domains *D*_*n *_and *D*_*r *_cooperatively interact with *D*_*m*_. Our approach for detecting cooperative domains in protein-protein interactions can be summarized as three steps. First, we extend the conventional probabilistic model for inferring domain interactions to accommodate multi-domain pairs. Then, the interaction probabilities of multi-domain pairs are estimated by the proposed approach. Finally, according to the interaction probabilities of multi-domain pairs, cooperative domains and superdomains are detected. The detailed information on the methodology is given in Methods.

### Identification of multi-domain cooperation

#### Identifying cooperative domains and superdomains

Our method is able to identify biologically meaningful superdomains and putative cooperative domains. We illustrate this feature by using MIPS data set. A cooperative domain pair has a stronger interaction effect than their corresponding two-domain pairs. Therefore, domains *D*_*m *_and *D*_*r *_are cooperative domains if Pr(*d*_*m*, *n *_= 1) *<*Pr(*d*_*mr*, *n *_= 1) and Pr(*d*_*r*, *n *_= 1) *<*Pr(*d*_*mr*, *n *_= 1) from the results of LPM or APMM. From the definitions, domains in a superdomain or in a strongly cooperative-domain pair are expected to have similar biological functions. We applied our approach to protein physical interaction data in MIPS1 (see Methods) to get reliable cooperative-domain interactions.

Totally we found 5187 two-domain interactions (with no-zero interaction probability), 83 superdomains and 650 cooperative-domain pairs, among which 525 pairs are strongly cooperative domain interactions according to the above definition. Some detected (strongly) cooperative domains and superdomains in MIPS1 are respectively listed in Tables 1, 2 and 3. To investigate functional relations of domains in these superdomains and cooperative domains, we listed their Pfam descriptions and GO annotations. GO similarity was computed for two domains both with GO annotations in superdomains (Table 1). From these tables, we can see that two domains in most superdomains and some cooperative domains have similar GO annotations or belong to a same family, which is consistent with our hypotheses, i.e., domains in a cooperative-domain interaction work cooperatively to facilitate specific functions. For instance, two domains in superdomains PF05000-PF00623, PF02775-PF00205, PF02800-PF00044 or PF00488–PF05192 have identical or similar functions at the respective GO levels. For those superdomains without GO annotations, the Pfam descriptions of two domains in most of them are also similar, such as PF08033-PF04810, PF03953-PF0009 or PF08544-PF00288. For cooperative domains, some of them have similar GO annotations of functions, such as PF00806-PF00076, where PF00806 is a Pumilio-family RNA binding repeat and PF00076 is a RNA recognition motif. Both domains are necessary for RNA binding. Some cooperative domains have no GO annotations but belong to same families, such as PF01466–PF03931. Both PF01466 (Skp1, dimerisation domain) and PF03931 (Skp1_POZ, tetramerisation domain) belong to the Skyp1 family. It is interesting that three domains in the strongly cooperative-domain interaction (PF04998-PF00623, PF01191) are found to have same functions, and they are all RNA polymerase domains. As another example, we identified cooperative domains PF00036–PF08226, and the Pfam description also supports the combination of PF08226 (DUF1720) with PF00036 (EF hand) [21]. Such facts imply that we can infer the functions of cooperative domains if one of them has known functions. We also applied our approach to DIP data set, and the detected superdomains and cooperative domains are given in Tables I-III (Additional File 1)

**Table 1:** Superdomains detected by our method from MIPS protein interaction data, where GO annotations are denoted in italic

Superdomains	Descriptions	GO similarity
PF00488, PF05192	(1) MutS domain V, *ATP binding, damaged DNA binding, mismatch repair* (2) MutS domain III, *DNA metabolism*	2-1-2-5-11-27-1-8-10 2-1-2-5-11-27-1 Similarity: 7
PF02775, PF00205	(1) Thiamine pyrophosphate enzyme, C-terminal TPP binding domain, *catalytic activity, thiamin pyrophosphate binding* (2) Thiamine pyrophosphate enzyme, central domain, *magnesium ion binding, thiamin pyrophosphate binding*	1-1-2-12-1-8 1-1-2-12-1-8 Similarity: 6
PF08033, PF04810	(1) Sec23/Sec24 beta-sandwich domain (2) Sec23/Sec24 zinc finger, COPII vesicle coat, *protein binding, intracellular protein transport, ER to Golgi vesicle-mediated transport*	
PF03953, PF00091	(1) Tubulin/FtsZ family, C-terminal domain, *protein complex, GTP binding, GTPase activity, protein polymerization* (2) Tubulin/FtsZ family, GTPase domain	
PF07687, PF01546	(1) Peptidase dimerisation domain, *hydrolase activity, protein dimerization activity* (2) Peptidase family M20/M25/M40, *metallopeptidase activity, proteolysis*	1-1-3-16 1-1-3-16-18-6 Similarity: 4
PF05000, PF00623	(1) RNA polymerase Rpb1, domain 4, *DNA-directed RNA polymerase activity, DNA binding, transcription* (2) RNA polymerase Rpb1, domain 2, *nucleus, DNA-directed RNA polymerase activity, DNA binding, transcription*	1-1-3-39-16-3-16 1-1-3-39-16-3-16 Similarity: 7
PF08544, PF00288	(1) GHMP kinases C terminal (2) GHMP kinases N terminal domain, *ATP binding, kinase activity, phosphorylation*	
PF01798, PF08060	(1) Putative snoRNA binding domain (2) NOSIC (NUC001) domain	
PF02800, PF00044	(1) Glyceraldehyde 3-phosphate dehydrogenase, C-terminal domain, *NAD binding, glyceraldehyde-3-phosphate dehydrogenase (phosphorylating) activity, glycolysis* (2) Glyceraldehyde 3-phosphate dehydrogenase, NAD binding domain, *NAD binding, glyceraldehyde-3-phosphate dehydrogenase (phosphorylating) activity, glycolysis*	2-1-2-5-11-1-4-9-1-5-1 2-1-2-5-11-1-4-9-1-5-1 Similarity: 11
PF08030, PF08022	(1) Ferric reductase NAD binding domain (2) FAD-binding domain	

**Table 2:** Cooperative domains detected by our method from MIPS protein interaction data, where GO annotations are denoted in italic

Cooperative domains (Interactor I)	Descriptions	Interactor II	Descriptions
PF00069, PF00786	(1) Protein kinase domain, *ATP binding, protein kinase activity, protein amino acid phosphorylation* (2) P21-Rho-binding domain	PF00018	SH3 domain, in a variety of proteins with enzymatic activity
PF00400, PF00646	(1) WD domain, G-beta repeat, coordinating multi-protein complex assemblies (2) F-box domain, mediating protein-protein interactions in a variety of contexts	PF01466	Skp1 family, dimerisation domain
PF00439, PF00176	(1) Bromodomain, interacting specifically with acetylated lysine (2) SNF2 family N-terminal domain, *DNA binding, ATP binding*	PF04433	SWIRM domain, mediating protein-protein interactions
PF00169, PF00787	(1) PH domain (2) PX domain, protein-protein interaction domain, *protein binding, phosphoinositide binding, intracellular signaling cascade*	PF08632	Sporulation protein Zds1 C terminal region, suppress the calcium sensitivity of Zds1 deletions
PF00069, PF00169	(1) Protein kinase domain, *ATP binding, protein kinase activity, protein amino acid phosphorylation* (2) PH domain	PF00018	SH3 domain, in a variety of proteins with enzymatic activity
PF00018, PF00063	(1) SH3 domain, in a variety of proteins with enzymatic activity (2) Myosin head (motor domain), myosin, *ATP binding, motor activity*	PF02205	WH2 motif, actin-binding motif
PF00806, PF00076	(1)Pumilio-family RNA binding repeat, *DNA binding* (2) RNA recognition motif	PF00501	AMP-binding enzyme, *catalytic activity, metabolism*
PF02985, PF03810	(1) HEAT repeat, involved in intracellular transport processes (2) Importin-beta N-terminal domain, *nuclear pore, nucleus, cytoplasm, protein transporter activity, protein import into nucleus, docking*	PF04096	Nucleoporin autopeptidase, *nuclear pore, transport*
PF02178, PF00271	(1) AT hook motif, DNA binding motifs (2) Helicase conserved C-terminal domain, *ATP binding, helicase activity, nucleic acid binding*	PF00249	Myb-like DNA-binding domain, *nucleus, DNA binding*

**Table 3:** Strongly cooperative domains detected by our method from MIPS protein interaction data, where GO annotations are denoted in italic

Cooperative domains (Interactor I)	Descriptions	Interactor II	Descriptions
PF00618, PF00018	(1) Guanine nucleotide exchange factor for Ras-like GTPases; N-terminal motif, *intracellular, regulation of small GTPase mediated signal transduction* (2) SH3 domain, in a variety of proteins with enzymatic activity	PF00012	Hsp70 protein, involved in different cellular compartments (nuclear, cytosolic, mitochondrial, endoplasmic reticulum, etc
PF01466, PF03931	(1) Skp1 family, dimerisation domain (2) Skp1 family, tetramerisation domain	PF00646	F-box domain, mediating protein-protein interactions
PF04998, PF00623	(1) RNA polymerase Rpb1, domain 5, *DNA-directed RNA polymerase activity, DNA binding, transcription* (2) RNA polymerase Rpb1, domain 2, nucleus, *DNA-directed RNA polymerase activity, DNA binding, transcription*	PF01191	RNA polymerase Rpb5, C-terminal domain, *DNA-directed RNA polymerase activity, DNA binding, transcription*
PF00806, PF00076	(1) Pumilio-family RNA binding repeat, *RNA binding* (2) RNA recognition motif, *nucleic acid binding*	PF00660	Seripauperin and TIP1 family, *response to stress*
PF00036, PF08226	(1) EF hand, *calcium ion binding* (2) Domain of unknown function (DUF1720), in different combinations with cortical patch components EF hand, SH3 and ENTH	PF07651	ANTH domain, *phospholipid binding*
PF00443, PF00581	(1) Ubiquitin carboxyl-terminal hydro-lase, *cysteine-type endopeptidase activity, ubiquitin thiolesterase activity, ubiquitin-dependent protein catabolism* (2) Rhodanese-like domain	PF00611	Fes/CIP4 homology domain, *regulatory processes*
PF00620, PF00787	(1) RhoGAP domain, *intracellular, signal transduction* (2) PX domain, *protein binding, phos-phoinositide binding, intracellular signaling cascade*	PF08632	Sporulation protein Zds1 C terminal region, sporulation, suppress the calcium sensitivity of Zds1 deletions
PF01426, PF00439	(1) BAH domain, DNA binding, involved in protein-protein interaction (2) bromodomain, involved in protein-protein interactions	PF00076	RNA recognition motif, *nucleic acid binding*

#### Verifying cooperative domains by crystal structures

In this section, we verify the detected cooperative domains by checking their physical interactions from the crystal structures of protein complexes in PDB and examine the essential mechanism of protein interactions at the domain level. The complex crystal structures in PDB can be regarded as a gold standard to verify protein interactions and domain interactions. The seq2struct web resource [26] was used to search sequence-structure links. By focusing on the protein pairs in which proteins are mapped to the same PDB IDs but possess different chain IDs, we found 50 protein pairs with crystal structures that contain cooperative-domain pairs identified by our approach (*t*-test value is significant by comparing with randomly generated domain pairs).

**Figure 2:**
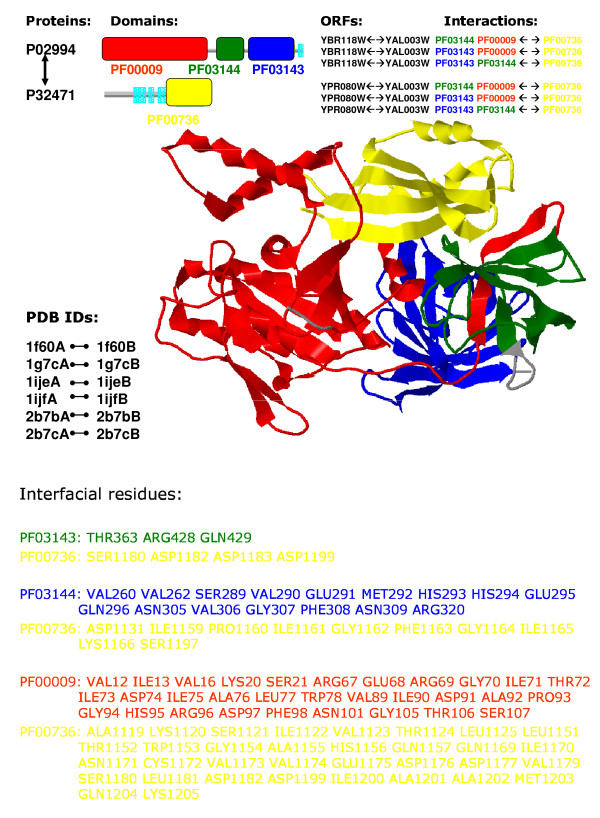
Cooperative domains in the complex crystal structure formed by proteins P02994 (with ORFs: YBR118W, YPR080W) and P32471 (with ORF: YAL003W). Protein sequences are shown using thick gray lines, and Pfam domain annotations are shown using colored rectangular boxes and drawn to scale (based on the Pfam database). The names of the protein sequences in this protein complex are listed to the upper left of the domain architecture. The identified cooperative domain pairs are listed to the upper right of the domain architecture. The domain names are labeled by the same color as in the Pfam domain annotation. The cartoon of PDB crystal structure (PDB ID: 1f60, Crystal structure of the yeast elongation factor complex) demonstrates the cooperative domain interactions (where domain colors are consistent with the domain annotation), i.e. domain PF00736 in protein P32471 interacts physically with domains of protein P02994. Other complexes in PDB containing these cooperative domains are also listed by their matched PDB IDs and chain IDs.

Figure 2 shows cooperative domains in a complex crystal structure formed by physical interactions of proteins P02994 (ORFs: YBR118W, YPR080W) and P32471 (ORF: YAL003W), where P02994 has three domains and P32471 has one domain PF00736. This complex is also included in the complex database PROTCOM [27]. Interacting protein pairs and their Pfam domain annotations are described in this figure. Clearly, EF-1 guanine nucleotide exchange domain PF00736 in P32471 has interactions with all of the domains in P02994. These interactions are verified by the binding sites of PF00736 with the domains in P02994. The cartoon of crystal structure illustrates that all cooperative-domain interactions in (P02994, P32471) are correctly identified and supported by the interfacial residues involved in the interaction. The interfacial residues are picked out by a simple rule, i.e. their C*α *atoms are within the distance threshold 10*Å*, which is consistent with the more accurate computation given in PROTCOM. This example provides an intuitive evidence for the cooperation among domains in the interaction of P02994 and P32471.

Furthermore we also revealed some complexes in PDB which are not reported by PROTCOM [27]. For example, three domains Arm, IBB and IBN_N which belong to Armadillo repeat superfamily form a cooperative-domain interaction (PF00514–PF01749, PF03810), and such multi-domain cooperation leads to the complex formed by protein Q02821 (YNL189W) and P33307 (YGL238W) (PDB ID 1wa5, GTP-Binding nuclear protein RAN). Generally, multiple cooperative-domain interactions in an interacting protein pair often correspond to a more complicated complex. The complete list of all the verified cooperative domain interactions by crystal structures in PDB and more detailed information are provided on our web site.

### PPI prediction based on multi-domain cooperation

#### Test on numerical PPI data sets

In addition to identifying superdomains and cooperative domains, our approach has a higher prediction accuracy for protein interactions by exploiting the information of both multi-domains and multiple organisms. In this section, we compared LPM and APMM with the existing methods, such as association based methods (ASNM [16], ASSOC [11]), and EM method [12]. Among those existing methods, the ASSOC and EM are developed for the binary interaction data whereas ASNM can be applied to experiment ratio data. We evaluated each method by fivefold cross validation on Ito's experiment ratio data [28] and assessed the prediction accuracy by root-mean-square error (RMSE) (see Methods).

The performance of each method in terms of RMSE and elapsed training time for fivefold cross-validation is summarized in Table 4. In order to check the effect of cooperative domains on the accuracy, here we computed RMSE only on those protein pairs containing cooperative-domain pairs. RMSE comparison results on all protein pairs are given in Table IV and Table V (Additional file 1). From Table 4 we can see that the performances of EM and ASSOC methods on experiment ratio data are not good since their training and testing errors are very high. ASNM which is an extension of ASSOC has much better results than ASSOC. LPM has a lower training and testing error than the methods based on two-domain pairs. Table 4 also indicates that APMM has the lowest error in both training and testing prediction of protein interactions. In addition, there is no significant increase on the computation time when multi-domain pairs are included.

**Table 4:** Comparisons of several methods in terms of RMSE and training time on Ito's dataset

	EM	ASSOC	ASNM	**LPM**	**APMM**
Train					
1st	0.4693	0.4537	0.0486	0.0084	0.0077
2nd	0.4810	0.4670	0.0486	0.0086	0.0079
3rd	0.4746	0.4617	0.0508	0.0071	0.0060
4th	0.4683	0.4545	0.0474	0.0076	0.0057
5th	0.4676	0.4540	0.0493	0.0072	0.0066
Average	0.4722	0.4582	0.0489	0.0073	0.0068
Time (seconds)	6.6622	0.0090	0.003	1.099	0.007

Test					
1st	0.6624	0.6072	0.0743	0.0224	0.0189
2nd	0.4880	0.4938	0.0531	0.0104	0.0128
3rd	0.5670	0.5338	0.0591	0.0425	0.0427
4th	0.5848	0.5745	0.0641	0.0296	0.0271
5th	0.6417	0.6308	0.0753	0.0354	0.0307
Average	0.5888	0.5680	0.0652	0.0281	0.0265

**Figure 3:**
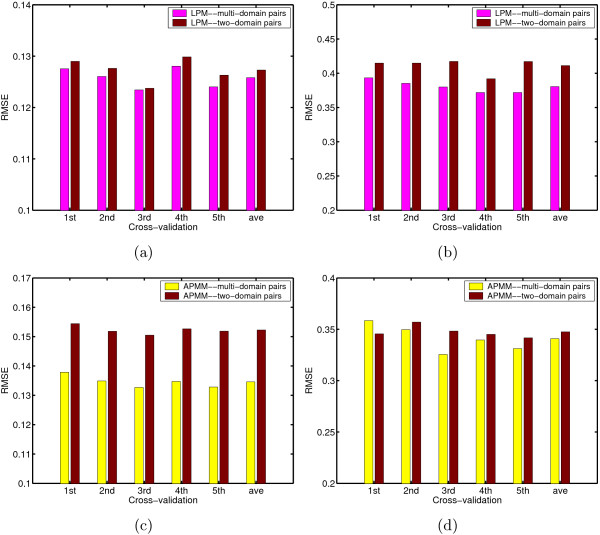
Comparisons of RMSE on two-domain pairs and on multiple-domain pairs for Krogan's yeast extended datasets. (a) The results of LPM on training. (b) The results of LPM on testing. (c) The results of APMM on training. (d) The results of APMM on testing.

The results for Ito's dataset in five rounds are not so consistent because there may exist bias in five divided subsets due to the small size of this dataset. Another larger dataset used for cross-validation is Krogan's confidence data [29] (see Methods). We used this set to test if or not various methods can correctly predict the interaction confidence of protein pairs. The result is summarized in Table 5, from which we can see that for the confidence prediction, our approach (LPM and APMM) employing multi-domain pairs again has better performance both in training and in testing than other methods. LPM generally has less training error than APMM but its testing error is slightly higher than that of APMM. An example to illustrate the effect of cooperative domains on the prediction accuracy is given in Additional file 1. We also made a direct comparison of our approach based on two-domain pairs and multi-domain pairs, and the results are summarized in Figure 3. We can see that LPM based on multi-domain pairs in training and testing has less prediction error in each round of fivefold cross validation than LPM based on only two-domain pairs. APMM also has such a tendency except the first round in testing. Such results further confirm the benefit of considering multi-domain interactions.

**Table 5:** Comparisons of of several methods in term of RMSE and training time on Krogan's yeast extended dataset

	EM	ASSOC	ASNM	**LPM**	**APMM**
Train					
1st	0.4156	0.4525	0.4580	0.1262	0.1359
2nd	0.4176	0.4521	0.4607	0.1248	0.1360
3rd	0.4196	0.4548	0.4615	0.1291	0.1365
4th	0.4178	0.4535	0.4585	0.1243	0.1337
5th	0.4184	0.4546	0.4602	0.1256	0.1338
Average	0.4178	0.4535	0.4598	0.1260	0.1352
Time (seconds)	2699.9	0.2000	0.1968	118.21	6.5092

Test					
1st	0.5504	0.5548	0.4931	0.3967	0.3588
2nd	0.5390	0.5441	0.4906	0.3804	0.3407
3rd	0.5372	0.5407	0.4822	0.3687	0.3372
4th	0.5422	0.5364	0.4805	0.3854	0.3366
5th	0.5333	0.5291	0.4747	0.3907	0.3455
Average	0.5404	0.5410	0.4842	0.3844	0.3437

#### Test on binary PPI data sets from multiple organisms

Compared with single organism, data sets from multiple organisms can provide more information, e.g. they cover more domains. In contrast to the existing methods which mainly use the data from single organism, LPM and APMM can employ the data sets from multiple organisms with the consideration of their different reliabilities. In this section, we used binary interaction data from multiple organisms collected by Liu et al. [14] (see Methods) to compare our approach with the extended EM algorithm [14] and validate the benefit of multi-domain pairs on binary interaction data.

**Figure 4:**
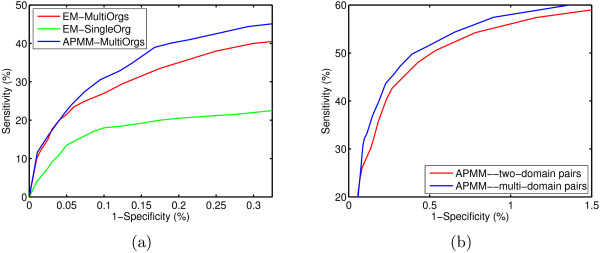
(a) ROC curve comparison of APMM and the extended EM on multiple-organism data. (b) ROC curve comparison of APMM based on two-domain pairs and multi-domain pairs.

Based on the same test set and training set, it is convenient to compare our methods and the extended EM [14]. The training sets respectively consist of protein interactions from single organism (yeast) and multiple organisms. Based on yeast protein interaction dataset, we found 4556 two-domain interactions (with no-zero interaction probability), 94 superdomains, 652 cooperative domain pairs, among which 640 pairs are strongly cooperative-domain interactions. In the protein interaction dataset of three organisms, we detected 34123 two-domain interactions (with no-zero interaction probability), 259 superdomains and 5633 cooperative-domain interactions, where 5400 are strongly cooperative. Among the cooperative-domain interactions in yeast and three organisms, 117 pairs are yeast-specific. With these domain interactions, the prediction accuracy of protein-protein interactions is measured by the receiver operating characteristic (ROC) curve, which is a plot of the true positive rate (sensitivity) against the false positive rate (1–specificity) for different thresholds. The result is plotted in Figure 4(a), from which we can see that APMM has a higher prediction accuracy than the extended EM algorithm on multiple-organism data. The AUC values of APMM, EM trained on multiple-organism data and EM trained on single-organism data are respectively 0.766, 0.701 and 0.611. This result indicates that APMM is also effective on binary interaction data. LPM has a similar performance as APMM which is not shown here.

To examine the effect of cooperative domains on prediction accuracy based on binary interaction data, we used the same training set and directly compared the prediction accuracies of APMM with multi-domain pairs and with only two-domain pairs. The result summarized in Figure 4(b) confirms that APMM with multi-domain pairs has a higher prediction accuracy. The performance of LPM was also confirmed in a similar manner.

### Evaluation at domain level and comparison with other methods

In this section, we evaluated our methods at the domain level by comparing the predicted domain interactions with domain interactions in iPfam [30] and the confidence DDI data in InterDom [31] (see Methods). The same data set in above section was used as training set.

The comparison results of the predicted domain interactions by LPM and APMM with those in iPfam are summarized in Table 6 with the significance of the overlap (*p*-value) computed by comparing with randomly predicted domain pairs (see Methods). We can see that domain interactions predicted by our approach have a significant overlap with iPfam and domain interactions predicted from multiple-organism data have larger overlap with iPfam owing to exploiting more information. Note that the overlap proportion is not so big. This is mainly because the amount of data in iPfam is highly incomplete and many domain interactions do not appear in iPfam.

**Table 6:** The overlap of the predicted domain interactions by APMM and LPM with those in iPfam, where λ denotes domain interaction probability, 'Single organism' means the training set of protein interactions is only from yeast, 'Multiple organisms' means the training set is from three organisms: yeast, worm and fly

Thresholds	Single organism (*p*-value)	Multiple Organisms (*p*-value)
APMM		
λ *>*0.1	110 (8.1e-009)	256 (8.4e-012)
λ *>*0.2	99 (*<*1e-013)	202 (2.9e-011)
λ *>*0.3	61 (9.8e-010)	149 (*<*1e-013)
λ *>*0.4	52 (3.2e-010)	127 (8.1-013)
λ *>*0.5	49 (1.5e-013)	91 (3.3e-012)

LPM		
λ *>*0.1	109 (2.1e-008)	256 (5.7e-013)
λ *>*0.2	97 (8.8e-013	201 (5.9e-013)
λ *>*0.3	61 (*<*1e-013)	148(2.9e-012)
λ *>*0.4	54 (4.4e-011)	130 (*<*1e-013)
λ *>*0.5	49 (*<*1e-013)	93 (*<*1e-013)

**Figure 5:**
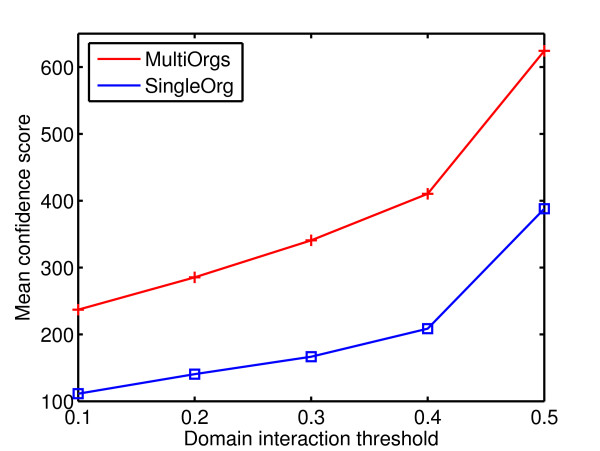
Mean confidence score of the predicted domain interactions (by APMM) at different domain interaction thresholds respectively based on single-organism data and multiple-organism data.

The comparison results of the predicted domain interactions by LPM and APMM with those in InterDom are summarized in Table 7 with *p*-values, which shows that the overlap with InterDom is significant and the interaction probabilities of predicted domain pairs are positively correlated with those in InterDom, i.e. a higher threshold corresponds to a higher mean confidence score. In addition, when selecting the same number of top high-scoring predicted domain interactions, the prediction result exploiting multiple-organism data has a much higher mean confidence score than that based on single-organism data, which is shown in Figure 5.

**Table 7:** The overlap of the predicted domain interactions by APMM and LPM with those in InterDom, where λ denotes domain interaction probability

Thresholds	Total domain pairs	InterDom overlap (*p*-value)	Mean significance
APMM			
λ *>*0.1	26407	8085 (8.0e-012)	75.3815
λ *>*0.2	16798	5834 (1.9e-011)	96.3991
λ *>*0.3	9416	3749 (*<*1e-013)	124.9704
λ *>*0.4	7582	3125 (1.5e-012)	140.3715
λ *>*0.5	4349	1800 (*<*1e-013)	170.5464

LPM			
λ *>*0.1	26326	8086 (1.7e-011)	75.3558
λ *>*0.2	16854	5844 (*<*1e-013)	96.6538
λ *>*0.3	9424	3753 (*<*1e-013)	124.8600
λ *>*0.4	7561	3101 (6.1e-012)	140.8661
λ *>*0.5	4322	1788 (1.3e-012)	171.6955

**Figure 6:**
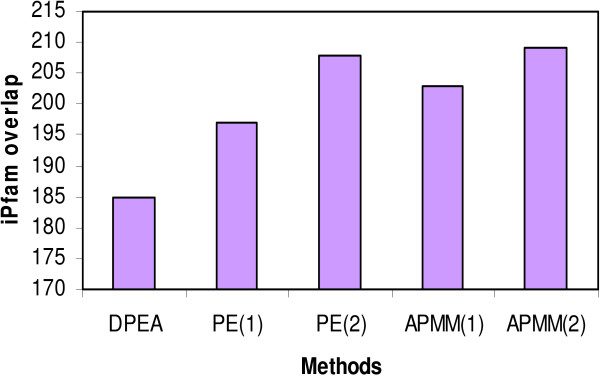
The overlaps of domain-domain interactions predicted by APMM, DPEA and PE with iPfam.

**Figure 7:**
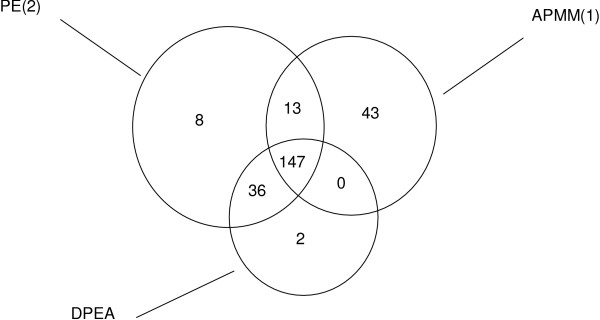
The distribution of the predicted DDI overlaps with iPfam by DPEA, PE and APMM.

The domain interactions in iPfam have been used as a gold standard set to evaluate the predicted domain-domain interactions [17,19]. We conducted a comparison experiment to evaluate the performance of our approach and the methods in Riley et al. [17] and Guimeraes et al. [19] based on the number of high-scoring domain-domain interactions confirmed by the gold standard set. Specifically, three methods (APMM in our work, DPEA in [17], PE in [19]) were applied in a same training set (DIP data, see Methods) and we checked the overlap of the predicted domain-domain interactions with iPfam by selecting a same number of high-scoring predicted domain interactions. The results based on 3005 high-scoring predicted domain interactions (provided by Riley et al. [17] and Guimeraes et al. [19]) are listed in Figure 6, where PE(1) denotes PE approach with network reliability 60% (LP-score ≥ 0.4, pw-score ≤ 0.1) and PE(2) denotes PE approach with network reliability 50% (LP-score ≥ 0.4, pw-score ≤ 0.1). APMM(1) means APMM based on multi-domain pairs and APMM(2) means APMM based on two-domain pairs. From Figure 6, we can see that our method has a comparable result with PE and a better performance than DPEA in terms of DDI prediction. Of course, this comparison result suffers from the incompleteness of the data in iPfam. Compared with APMM based on two-domain pairs, APMM based on multi-domain pairs has a slightly smaller iPfam overlap. This is because when our methods include multi-domain pairs, according to the definition of cooperative domains and the rule of selecting variables, some two-domain pairs are replaced with cooperative-domain pairs. At the same time, the current gold standard set only contains two-domain interactions. The distribution of the iPfam overlaps is shown in Figure 7 which indicates that our method can be an important complement since there is a large proportion of predicted DDIs not covered by other methods. Note that except two-domain interactions, our method can also infer cooperative-domain interactions.

As for identifying cooperative domains, we made a rough comparison with Han et al.'s domain combination approach [22,23]. Han et al. provided on their website [32] a set of 5826 proteins based on which they listed 300 predicted domain combination interacting pairs with top confidence. Among these 300 pairs, there are 246 two-domain pairs, 44 three-domain pairs and 10 four and above domain pairs. We applied our approach to the same dataset and found 34 cooperative-domain pairs (with interaction probability 1.0) among 500 high-scoring domain interactions, 225 among 1000 high-scoring domain interactions, and 635 among 2000 high-scoring domain interactions. Note that in 500 high-scoring domain interactions, the number of cooperative domains is not more than Han's (34/500*<*44/300), but when the threshold is lower, we found more cooperative domains. This is mainly due to the difference between the definitions of cooperative domains and domain combinations. In our approach, if a two-domain pair has a stronger interaction, the three-domain pair in the same protein pair will not be included as potential cooperative domains. If two-domain pairs have weaker interactions than the corresponding cooperative-domain interaction, this three-domain pair will be considered and the two-domain pairs will be excluded in the same protein pair. In other words, we eliminate the redundancy between domain combinations, whereas in Han et al.'s method, each domain combination is included.

## Discussion

In this work, cooperative domains, strongly cooperative domains and superdomains in MIPS and DIP were detected, and many of them were verified by the crystal structures in PDB. Functional relations in superdomains and cooperative domains were examined by the terms of GO. Among the detected superdomains, we found that two domains in most of them belong to a same family and have similar or identical functions. Such fact is biologically reasonable because the two domains in a superdomain always appear together in individual proteins and participate in interaction processes simultaneously. It is interesting that many domains that act as superdomains are binding domains, such as snoRNA binding, ATP binding, DNA binding, FAD-binding, NAD-binding, GTP binding, protein binding, and Lum-binding. For cooperative domains, some of them have dissimilar functions partly because domain cooperation needs complementary functions [33]. Cooperative domains tend to be contained in big complicated protein complexes. For example, among cooperative domains with crystal structures, 72% of (36/50) them are involved in large complexes with more than five proteins. This fact to some extent illustrates that multi-domain cooperation can be easily achieved in a multi-protein complex where protein cooperation is prominent.

Multi-domain cooperation information can be explored to reconstruct the structure of a large protein complex. Protein complexes are key molecular entities that integrate multiple gene products to perform cellular functions. Recently, tandem-affinity-purification coupled to mass spectrometry (TAP-MS) which combines affinity tags-based protein purification technique and mass spectrometry for identifying a tagged protein and its interaction partners [34] has been applied to find the genome-wide screen for complexes [29,35]. However, although many complexes have now been identified, the detailed interacting relationships among the components are beyond our knowledge because only a few of them have 3D structural information. As pointed in Aloy and Russell [36], X-ray crystallography provides atomic-resolution models for proteins and complexes, but it is difficult for this technique to obtain sufficient information for the crystallization of large complexes. NMR is generally limited to proteins that have no more than 300 residues. It is therefore necessary and timely to develop new approaches that can reconstruct the structures of complexes based on protein structures and their interaction relationships [37]. The detected cooperative domains in this work can be applied to this problem by combining with docking procedures.

**Figure 8:**
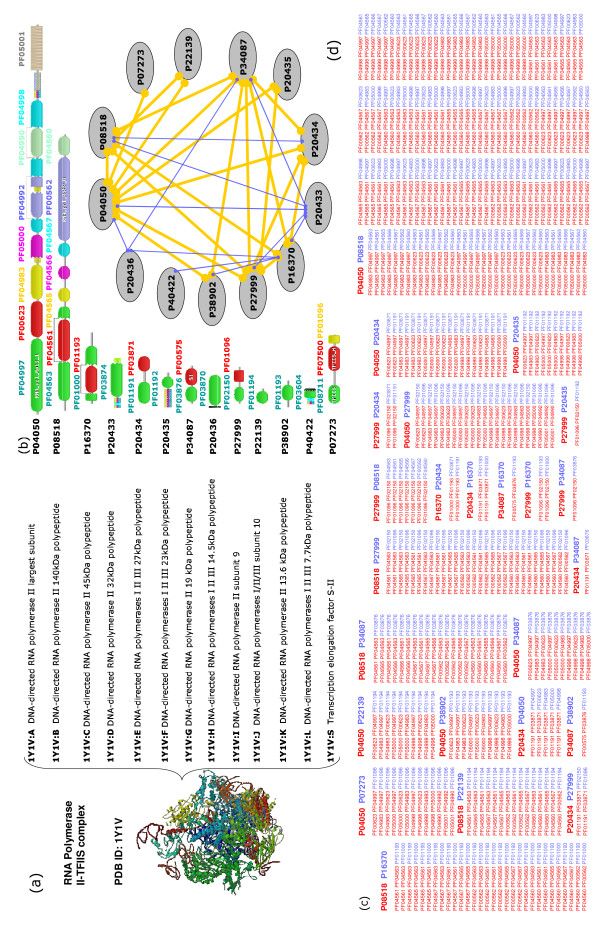
Reconstruction of DNA-directed RNA polymerase complex. (a) The RNA Polymerase II-TFIIS complex (PDB ID 1y1v) with 13 subunits (from chain A to chain S). Every chain is one protein (shown by their UniProtKB accessions) and their complex interactions form the large polymer. (b) The PfamA domain architecture for every protein. (c) The cooperative domains identified by our method with protein interaction pairs containing them. The red or blue colors of proteins and domains indicate their memberships.

As an example, Figure 8 illustrates how to reconstruct the RNA Polymerase II-TFIIS complex (PDB ID 1y1v) by our approach at protein, domain and atomic levels respectively with the following five steps. In the first step, from the information of TAP-MS, there are total 13 different proteins (P04050, P08518, P16370, P20433, P20434, P20435, P34087, P20436, P27999, P22139, P38902, P40422, P07273) in this complex as its subunits (Figure 8(a)). Then, in the second step, according to Pfam and protein sequences, all possible domains for each protein in terms of Pfam architectures can be obtained (Figure 8(b)). There are 30 domains involved in this complex, so it is infeasible to clearly explain the interaction relationships between domains and proteins only by two-domain pairs (total 1435 pairs). In the third step, cooperative domain interactions are obtained by performing our approach on protein interaction data, which provide valuable information (Figure 8(c)). In the fourth step, physical interactions of those 13 proteins in the complex can be predicted at the protein level (see Figure 8(d), where thick lines denote the physical interactions realized by cooperative domain interactions and thin ones are realized by two-domain interactions). In the fifth step, the interactions between protein pairs are further examined at the domain levels based on complex structure information. For example, by examining the domain interactions between proteins P08518 (seven domains) and P16370 (two domains), we found that all of domains in the two proteins cooperatively interact with other domains except PF04566, as shown by a 3D structure from PDB in Figure I (Additional file 1). On the other hand, it is easy to estimate the interaction relations between proteins by considering cooperative domains. For example, proteins P04050 and P08518 probably have the strongest interaction because they have many cooperative-domain interactions. By further combining our method with a protein docking procedure (for searching the interacting areas of domains) [38], the detailed interactions at atomic level can be identified, which makes it possible to construct the stable and coherent crystal structure of a complex.

## Conclusion

Domains are viewed as the basic functional units of proteins, and it is believed that protein interactions are achieved through domain interactions. Most existing methods for inferring protein interactions from experimental data assume that two-domain pairs are dominating factors for protein interactions. However, like the cooperation of several proteins in a complex, many domains may be cooperative in achieving the interaction of a protein pair. In this paper, we focus on revealing such domain cooperation by considering multi-domain pairs as the basic units of protein interactions. In addition, in contrast to the existing methods which mainly use the data from single organism, data sets for multiple species with different reliabilities were exploited in this paper to make full use of the available information. From the computational viewpoint, this paper provides a general framework based on APMM and LPM to predict protein interactions in a more accurate manner by considering the information of both multi-domain pairs and multiple organisms, which can also be applied to identify cooperative domains. Experiment results demonstrated that our method not only can find superdomains and putative cooperative domains effectively but also has a higher prediction accuracy of protein interactions than the existing methods. Cooperative domains, strongly cooperative domains and superdomains in MIPS and DIP were detected, and many of them were verified by the crystal structures in PDB. Comparison experiments on protein/domain interaction prediction confirm the benefit of considering multi-domain cooperation. More detailed results and software can be found at our website.

## Methods

### Data sources

In this work, we validated our approach using several types of experiments which employed various experiment data sets as follows.

#### Binary PPI data

When we test our method for detecting cooperative domains and PPI prediction based on multiple-organism data, we used binary interaction data in which the information is whether two proteins interact or not. In other words, there is not a confidence score for each protein-protein interaction. We collected 4103 physical interactions in yeast from MIPS [4] (the version is PPI_141105.tab, denoted as MIPS1) and identified superdomains and cooperative domains in this dataset.

For PPI prediction based on multiple-organism data, in order to make comparison convenient, we used the same training data and testing data collected by Liu et al. [14]. These datasets are from yeast *S.cerevisiae*, worm *C.elegans *and fly *D.melanogaster *with respectively 5295, 4714 and 20349 protein interactions. The protein-domain relationships for each protein are extracted from PFAM [21] and SMART [39]. Among these protein interaction data, only those with domain information were used. In addition, like in Liu et al. [14], an independent test set including the 3543 yeast physical interaction pairs in MIPS (denoted it as MIPS2) was used as positive examples and the other possible protein pairs, totally 6895215 pairs, as negative examples.

For comparison experiments at the domain interaction level, we used protein-protein interactions and protein domain composition dataset in Riley et al. [17] and Guimaraes et al. [19]. This set was obtained from the DIP database [3] and contains 26,032 interactions underlying 11,403 proteins from 69 organisms.

#### Numerical PPI data

Numerical interaction data are defined as opposite to binary interaction data. It means that each protein-protein interaction has a score to denote the interaction strength. It includes experiment ratio data based on IST [28] and confidence data by integrating various data sources [29]. IST (Interaction Sequence Tags) was used for decoding interacting proteins in examining two-hybrid interactions. Experiment ratio data based on IST mean that each protein-protein interaction is provided with the number of IST hitting in a certain number of experiments. We conducted cross-validation experiment on numerical interaction data. The first set is a well known dataset– the full data of Ito's dataset [28]. This dataset has 1586 interactions with 1420 proteins containing domain information, and provides the numerical interaction (ratio) data for protein pairs based on the number of IST hits. The other is Krogan's extended dataset [29]. This set has 10265 interactions with 2843 proteins containing domain information. It provides each protein interaction with a confidence score.

#### Domain sources and DDI data

The domain information for proteins was extracted from Pfam 14.0 [21]. For MIPS1, there are total 1483 Pfam domains involved in 2477 proteins. iPfam database [30] contains domain-domain interactions confirmed by PDB crystal structures. It has been used as a gold standard set for evaluating predicted domain-domain interactions [17,19]. In this work, 3034 domain interactions in iPfam (December 2005 version) were used for evaluating domain interaction prediction. In addition, InterDom (version 1.2) [31] was also used for this purpose. It is a database of putative interacting domains derived from multiple data sources, ranging from domain fusions (Rosetta Stone), protein interactions (DIP and BIND), protein complexes (PDB), to scientific literature (MEDLINE). InterDom 1.2 has 30038 putative domain interactions with different confidence scores.

#### Protein complex and crystal structure database

Cooperative domains were confirmed using structural data of protein complexes from PDB and PROTOCOM. Protein sequences were mapped from Swiss-Prot/TrEMBL database to their corresponding structure files using the seq2struct web resource [26]. PROTOCOM [27] is a collection of protein-protein transient complexes and domain-domain structures. It provides the detailed information about protein interactions by identifying the contacted residues, presenting the number of residues on the interface and the list of interfacial residues.

### Probabilistic model with multi-domain pairs

In this section, we describe an improved probabilistic model for protein interactions by considering the multi-domain pairs, which is the essential basis of our method.

Assume that in the protein interactions of *K *species (or data sets), there are *N*_*k *_proteins in dataset *k *respectively denoted by P1K
 MathType@MTEF@5@5@+=feaafiart1ev1aaatCvAUfKttLearuWrP9MDH5MBPbIqV92AaeXatLxBI9gBaebbnrfifHhDYfgasaacH8akY=wiFfYdH8Gipec8Eeeu0xXdbba9frFj0=OqFfea0dXdd9vqai=hGuQ8kuc9pgc9s8qqaq=dirpe0xb9q8qiLsFr0=vr0=vr0dc8meaabaqaciaacaGaaeqabaqabeGadaaakeaacqWGqbaudaqhaaWcbaGaeGymaedabaGaem4saSeaaaaa@3011@, …, PNkk
 MathType@MTEF@5@5@+=feaafiart1ev1aaatCvAUfKttLearuWrP9MDH5MBPbIqV92AaeXatLxBI9gBaebbnrfifHhDYfgasaacH8akY=wiFfYdH8Gipec8Eeeu0xXdbba9frFj0=OqFfea0dXdd9vqai=hGuQ8kuc9pgc9s8qqaq=dirpe0xb9q8qiLsFr0=vr0=vr0dc8meaabaqaciaacaGaaeqabaqabeGadaaakeaacqWGqbaudaqhaaWcbaGaemOta40aaSbaaWqaaiabdUgaRbqabaaaleaacqWGRbWAaaaaaa@321D@*k *= 1, …, *K*, with *M *domains in all of these proteins represented by *D*_1_, …, *D*_*M *_. Let Pik
 MathType@MTEF@5@5@+=feaafiart1ev1aaatCvAUfKttLearuWrP9MDH5MBPbIqV92AaeXatLxBI9gBaebbnrfifHhDYfgasaacH8akY=wiFfYdH8Gipec8Eeeu0xXdbba9frFj0=OqFfea0dXdd9vqai=hGuQ8kuc9pgc9s8qqaq=dirpe0xb9q8qiLsFr0=vr0=vr0dc8meaabaqaciaacaGaaeqabaqabeGadaaakeaacqWGqbaudaqhaaWcbaGaemyAaKgabaGaem4AaSgaaaaa@30BC@ also denote a set of domains in the protein *i *of dataset *k*. Define Pijk
 MathType@MTEF@5@5@+=feaafiart1ev1aaatCvAUfKttLearuWrP9MDH5MBPbIqV92AaeXatLxBI9gBaebbnrfifHhDYfgasaacH8akY=wiFfYdH8Gipec8Eeeu0xXdbba9frFj0=OqFfea0dXdd9vqai=hGuQ8kuc9pgc9s8qqaq=dirpe0xb9q8qiLsFr0=vr0=vr0dc8meaabaqaciaacaGaaeqabaqabeGadaaakeaacqWGqbaudaqhaaWcbaGaemyAaKMaemOAaOgabaGaem4AaSgaaaaa@3219@ to represent a protein pair (Pik
 MathType@MTEF@5@5@+=feaafiart1ev1aaatCvAUfKttLearuWrP9MDH5MBPbIqV92AaeXatLxBI9gBaebbnrfifHhDYfgasaacH8akY=wiFfYdH8Gipec8Eeeu0xXdbba9frFj0=OqFfea0dXdd9vqai=hGuQ8kuc9pgc9s8qqaq=dirpe0xb9q8qiLsFr0=vr0=vr0dc8meaabaqaciaacaGaaeqabaqabeGadaaakeaacqWGqbaudaqhaaWcbaGaemyAaKgabaGaem4AaSgaaaaa@30BC@, Pjk
 MathType@MTEF@5@5@+=feaafiart1ev1aaatCvAUfKttLearuWrP9MDH5MBPbIqV92AaeXatLxBI9gBaebbnrfifHhDYfgasaacH8akY=wiFfYdH8Gipec8Eeeu0xXdbba9frFj0=OqFfea0dXdd9vqai=hGuQ8kuc9pgc9s8qqaq=dirpe0xb9q8qiLsFr0=vr0=vr0dc8meaabaqaciaacaGaaeqabaqabeGadaaakeaacqWGqbaudaqhaaWcbaGaemOAaOgabaGaem4AaSgaaaaa@30BE@) and *D*_*m, n *_to represent a domain pair (*D*_*m*_, *D*_*n*_). We also introduce a symbol *D*_*m*, *rn *_for a cooperative-domain pair (*D*_*m *_- *D*_*r*_, *D*_*n*_) to represent the case that domains *D*_*m *_and *D*_*r *_in protein Pik
 MathType@MTEF@5@5@+=feaafiart1ev1aaatCvAUfKttLearuWrP9MDH5MBPbIqV92AaeXatLxBI9gBaebbnrfifHhDYfgasaacH8akY=wiFfYdH8Gipec8Eeeu0xXdbba9frFj0=OqFfea0dXdd9vqai=hGuQ8kuc9pgc9s8qqaq=dirpe0xb9q8qiLsFr0=vr0=vr0dc8meaabaqaciaacaGaaeqabaqabeGadaaakeaacqWGqbaudaqhaaWcbaGaemyAaKgabaGaem4AaSgaaaaa@30BC@ cooperatively interact with domain *D*_*n *_in protein Pjk
 MathType@MTEF@5@5@+=feaafiart1ev1aaatCvAUfKttLearuWrP9MDH5MBPbIqV92AaeXatLxBI9gBaebbnrfifHhDYfgasaacH8akY=wiFfYdH8Gipec8Eeeu0xXdbba9frFj0=OqFfea0dXdd9vqai=hGuQ8kuc9pgc9s8qqaq=dirpe0xb9q8qiLsFr0=vr0=vr0dc8meaabaqaciaacaGaaeqabaqabeGadaaakeaacqWGqbaudaqhaaWcbaGaemOAaOgabaGaem4AaSgaaaaa@30BE@. *D*_*m*, *rn *_has a similar implication. In our probabilistic model, Pijk
 MathType@MTEF@5@5@+=feaafiart1ev1aaatCvAUfKttLearuWrP9MDH5MBPbIqV92AaeXatLxBI9gBaebbnrfifHhDYfgasaacH8akY=wiFfYdH8Gipec8Eeeu0xXdbba9frFj0=OqFfea0dXdd9vqai=hGuQ8kuc9pgc9s8qqaq=dirpe0xb9q8qiLsFr0=vr0=vr0dc8meaabaqaciaacaGaaeqabaqabeGadaaakeaacqWGqbaudaqhaaWcbaGaemyAaKMaemOAaOgabaGaem4AaSgaaaaa@3219@ is also used to represent the set of domain pairs including all multi-domain pairs in Pik
 MathType@MTEF@5@5@+=feaafiart1ev1aaatCvAUfKttLearuWrP9MDH5MBPbIqV92AaeXatLxBI9gBaebbnrfifHhDYfgasaacH8akY=wiFfYdH8Gipec8Eeeu0xXdbba9frFj0=OqFfea0dXdd9vqai=hGuQ8kuc9pgc9s8qqaq=dirpe0xb9q8qiLsFr0=vr0=vr0dc8meaabaqaciaacaGaaeqabaqabeGadaaakeaacqWGqbaudaqhaaWcbaGaemyAaKgabaGaem4AaSgaaaaa@30BC@, and Pjk
 MathType@MTEF@5@5@+=feaafiart1ev1aaatCvAUfKttLearuWrP9MDH5MBPbIqV92AaeXatLxBI9gBaebbnrfifHhDYfgasaacH8akY=wiFfYdH8Gipec8Eeeu0xXdbba9frFj0=OqFfea0dXdd9vqai=hGuQ8kuc9pgc9s8qqaq=dirpe0xb9q8qiLsFr0=vr0=vr0dc8meaabaqaciaacaGaaeqabaqabeGadaaakeaacqWGqbaudaqhaaWcbaGaemOAaOgabaGaem4AaSgaaaaa@30BE@ i.e.,

Pijk={Dm,n|Dm∈Pik,Dn∈Pjk}∪{Dmr,n|Dm,Dr∈Pik,Dn∈Pjk}∪{Dm,nr|Dm∈Pik,Dn,Dr∈Pjk}.
 MathType@MTEF@5@5@+=feaafiart1ev1aaatCvAUfKttLearuWrP9MDH5MBPbIqV92AaeXatLxBI9gBaebbnrfifHhDYfgasaacH8akY=wiFfYdH8Gipec8Eeeu0xXdbba9frFj0=OqFfea0dXdd9vqai=hGuQ8kuc9pgc9s8qqaq=dirpe0xb9q8qiLsFr0=vr0=vr0dc8meaabaqaciaacaGaaeqabaqabeGadaaakeaacqWGqbaudaqhaaWcbaGaemyAaKMaemOAaOgabaGaem4AaSgaaOGaeyypa0Jaei4EaSNaemiraq0aaSbaaSqaaiabd2gaTjabcYcaSiabd6gaUbqabaGccqGG8baFcqWGebardaWgaaWcbaGaemyBa0gabeaakiabgIGiolabdcfaqnaaDaaaleaacqWGPbqAaeaacqWGRbWAaaGccqGGSaalcqWGebardaWgaaWcbaGaemOBa4gabeaakiabgIGiolabdcfaqnaaDaaaleaacqWGQbGAaeaacqWGRbWAaaGccqGG9bqFcqWIQisvcqGG7bWEcqWGebardaWgaaWcbaGaemyBa0MaemOCaiNaeiilaWIaemOBa4gabeaakiabcYha8jabdseaenaaBaaaleaacqWGTbqBaeqaaOGaeiilaWIaemiraq0aaSbaaSqaaiabdkhaYbqabaGccqGHiiIZcqWGqbaudaqhaaWcbaGaemyAaKgabaGaem4AaSgaaOGaeiilaWIaemiraq0aaSbaaSqaaiabd6gaUbqabaGccqGHiiIZcqWGqbaudaqhaaWcbaGaemOAaOgabaGaem4AaSgaaOGaeiyFa0NaeSOkIuLaei4EaSNaemiraq0aaSbaaSqaaiabd2gaTjabcYcaSiabd6gaUjabdkhaYbqabaGccqGG8baFcqWGebardaWgaaWcbaGaemyBa0gabeaakiabgIGiolabdcfaqnaaDaaaleaacqWGPbqAaeaacqWGRbWAaaGccqGGSaalcqWGebardaWgaaWcbaGaemOBa4gabeaakiabcYcaSiabdseaenaaBaaaleaacqWGYbGCaeqaaOGaeyicI4Saemiuaa1aa0baaSqaaiabdQgaQbqaaiabdUgaRbaakiabc2ha9jabc6caUaaa@911C@

Let the interaction between Pik
 MathType@MTEF@5@5@+=feaafiart1ev1aaatCvAUfKttLearuWrP9MDH5MBPbIqV92AaeXatLxBI9gBaebbnrfifHhDYfgasaacH8akY=wiFfYdH8Gipec8Eeeu0xXdbba9frFj0=OqFfea0dXdd9vqai=hGuQ8kuc9pgc9s8qqaq=dirpe0xb9q8qiLsFr0=vr0=vr0dc8meaabaqaciaacaGaaeqabaqabeGadaaakeaacqWGqbaudaqhaaWcbaGaemyAaKgabaGaem4AaSgaaaaa@30BC@ and Pjk
 MathType@MTEF@5@5@+=feaafiart1ev1aaatCvAUfKttLearuWrP9MDH5MBPbIqV92AaeXatLxBI9gBaebbnrfifHhDYfgasaacH8akY=wiFfYdH8Gipec8Eeeu0xXdbba9frFj0=OqFfea0dXdd9vqai=hGuQ8kuc9pgc9s8qqaq=dirpe0xb9q8qiLsFr0=vr0=vr0dc8meaabaqaciaacaGaaeqabaqabeGadaaakeaacqWGqbaudaqhaaWcbaGaemOAaOgabaGaem4AaSgaaaaa@30BE@ (between *D*_*m *_and *D*_*n*_) be represented by a random variable pijk
 MathType@MTEF@5@5@+=feaafiart1ev1aaatCvAUfKttLearuWrP9MDH5MBPbIqV92AaeXatLxBI9gBaebbnrfifHhDYfgasaacH8akY=wiFfYdH8Gipec8Eeeu0xXdbba9frFj0=OqFfea0dXdd9vqai=hGuQ8kuc9pgc9s8qqaq=dirpe0xb9q8qiLsFr0=vr0=vr0dc8meaabaqaciaacaGaaeqabaqabeGadaaakeaacqWGWbaCdaqhaaWcbaGaemyAaKMaemOAaOgabaGaem4AaSgaaaaa@3259@ (*d*_*m*, *n*_). Accordingly we introduce random variables *d*_*mr*, *n *_to denote whether domains *D*_*m *_and *D*_*r *_cooperatively interact with domain *D*_*n *_or not. The probabilistic model [12,13] for inferring protein interactions has two basic assumptions. One is that domain interactions in each protein pair are independent. The other is that two proteins interact if and only if there is at least one interacting domain pair in this protein pair. In the improved model, we also make these assumptions, but extend two-domain interactions to multi-domain interactions. Therefore, the interaction probability of Pik
 MathType@MTEF@5@5@+=feaafiart1ev1aaatCvAUfKttLearuWrP9MDH5MBPbIqV92AaeXatLxBI9gBaebbnrfifHhDYfgasaacH8akY=wiFfYdH8Gipec8Eeeu0xXdbba9frFj0=OqFfea0dXdd9vqai=hGuQ8kuc9pgc9s8qqaq=dirpe0xb9q8qiLsFr0=vr0=vr0dc8meaabaqaciaacaGaaeqabaqabeGadaaakeaacqWGqbaudaqhaaWcbaGaemyAaKgabaGaem4AaSgaaaaa@30BC@ and Pjk
 MathType@MTEF@5@5@+=feaafiart1ev1aaatCvAUfKttLearuWrP9MDH5MBPbIqV92AaeXatLxBI9gBaebbnrfifHhDYfgasaacH8akY=wiFfYdH8Gipec8Eeeu0xXdbba9frFj0=OqFfea0dXdd9vqai=hGuQ8kuc9pgc9s8qqaq=dirpe0xb9q8qiLsFr0=vr0=vr0dc8meaabaqaciaacaGaaeqabaqabeGadaaakeaacqWGqbaudaqhaaWcbaGaemOAaOgabaGaem4AaSgaaaaa@30BE@ is given by

Pr⁡(pijk=1)=1−∏Dm,n∈Pijk(1−Pr⁡(dm,n=1))∏Dmr,n∈Pijk(1−Pr⁡(dmr,n=1))∏Dm,nr∈Pijk(1−Pr⁡(dm,nr=1))
 MathType@MTEF@5@5@+=feaafiart1ev1aaatCvAUfKttLearuWrP9MDH5MBPbIqV92AaeXatLxBI9gBaebbnrfifHhDYfgasaacH8akY=wiFfYdH8Gipec8Eeeu0xXdbba9frFj0=OqFfea0dXdd9vqai=hGuQ8kuc9pgc9s8qqaq=dirpe0xb9q8qiLsFr0=vr0=vr0dc8meaabaqaciaacaGaaeqabaqabeGadaaakeaacyGGqbaucqGGYbGCcqGGOaakcqWGWbaCdaqhaaWcbaGaemyAaKMaemOAaOgabaGaem4AaSgaaOGaeyypa0JaeGymaeJaeiykaKIaeyypa0JaeGymaeJaeyOeI0YaaebuaeaacqGGOaakcqaIXaqmcqGHsislcyGGqbaucqGGYbGCcqGGOaakcqWGKbazdaWgaaWcbaGaemyBa0MaeiilaWIaemOBa4gabeaakiabg2da9iabigdaXiabcMcaPiabcMcaPaWcbaGaemiraq0aaSbaaWqaaiabd2gaTjabcYcaSiabd6gaUbqabaWccqGHiiIZcqWGqbaudaqhaaadbaGaemyAaKMaemOAaOgabaGaem4AaSgaaaWcbeqdcqGHpis1aOWaaebuaeaacqGGOaakcqaIXaqmcqGHsislcyGGqbaucqGGYbGCcqGGOaakcqWGKbazdaWgaaWcbaGaemyBa0MaemOCaiNaeiilaWIaemOBa4gabeaakiabg2da9iabigdaXiabcMcaPiabcMcaPmaarafabaGaeiikaGIaeGymaeJaeyOeI0IagiiuaaLaeiOCaiNaeiikaGIaemizaq2aaSbaaSqaaiabd2gaTjabcYcaSiabd6gaUjabdkhaYbqabaGccqGH9aqpcqaIXaqmcqGGPaqkcqGGPaqkaSqaaiabdseaenaaBaaameaacqWGTbqBcqGGSaalcqWGUbGBcqWGYbGCaeqaaSGaeyicI4Saemiuaa1aa0baaWqaaiabdMgaPjabdQgaQbqaaiabdUgaRbaaaSqab0Gaey4dIunaaSqaaiabdseaenaaBaaameaacqWGTbqBcqWGYbGCcqGGSaalcqWGUbGBaeqaaSGaeyicI4Saemiuaa1aa0baaWqaaiabdMgaPjabdQgaQbqaaiabdUgaRbaaaSqab0Gaey4dIunaaaa@9836@

where Pr(pijk
 MathType@MTEF@5@5@+=feaafiart1ev1aaatCvAUfKttLearuWrP9MDH5MBPbIqV92AaeXatLxBI9gBaebbnrfifHhDYfgasaacH8akY=wiFfYdH8Gipec8Eeeu0xXdbba9frFj0=OqFfea0dXdd9vqai=hGuQ8kuc9pgc9s8qqaq=dirpe0xb9q8qiLsFr0=vr0=vr0dc8meaabaqaciaacaGaaeqabaqabeGadaaakeaacqWGWbaCdaqhaaWcbaGaemyAaKMaemOAaOgabaGaem4AaSgaaaaa@3259@ = 1) represents the interaction probability of proteins Pik
 MathType@MTEF@5@5@+=feaafiart1ev1aaatCvAUfKttLearuWrP9MDH5MBPbIqV92AaeXatLxBI9gBaebbnrfifHhDYfgasaacH8akY=wiFfYdH8Gipec8Eeeu0xXdbba9frFj0=OqFfea0dXdd9vqai=hGuQ8kuc9pgc9s8qqaq=dirpe0xb9q8qiLsFr0=vr0=vr0dc8meaabaqaciaacaGaaeqabaqabeGadaaakeaacqWGqbaudaqhaaWcbaGaemyAaKgabaGaem4AaSgaaaaa@30BC@ and Pjk
 MathType@MTEF@5@5@+=feaafiart1ev1aaatCvAUfKttLearuWrP9MDH5MBPbIqV92AaeXatLxBI9gBaebbnrfifHhDYfgasaacH8akY=wiFfYdH8Gipec8Eeeu0xXdbba9frFj0=OqFfea0dXdd9vqai=hGuQ8kuc9pgc9s8qqaq=dirpe0xb9q8qiLsFr0=vr0=vr0dc8meaabaqaciaacaGaaeqabaqabeGadaaakeaacqWGqbaudaqhaaWcbaGaemOAaOgabaGaem4AaSgaaaaa@30BE@ in dataset *k*, and Pr(*d*_*m*, *n *_= 1) represents the probability that domain *D*_*m *_interacts with *D*_*n*_. Pr(*d*_*mr*, *n *_= 1) represents the probability that domains *D*_*m *_and *D*_*r *_cooperatively interact with *D*_*n*_. Pr(*d*_*m*, *nr *_= 1) has a similar meaning. For each protein pair in (1), if there is a cooperative interaction of domains *D*_*m *_- *D*_*r *_with domain *D*_*n *_in the second multiplying term, then (*D*_*m*_, *D*_*n*_) and (*D*_*r*_, *D*_*n*_) must be excluded from the first multiplying term in order to maintain the independence assumption; otherwise, (*D*_*m *_- *D*_*r*_, *D*_*n*_) should be deleted. The third multiplying term for (*D*_*m*_, *D*_*r *_- *D*_*n*_) should be checked in the same way. Clearly, the first multiplying term represents the effect of two-domain pair interactions while the second and third multiplying terms stand for the effects of cooperative-domain interactions. In next section, we will show how to determine those independent variables.

Note that we extend two-domain interactions only to three-domain interactions because the cooperation involving more than three domains is believed to be rare compared with cases of two and three domains, though theoretically model (1) can be further extended to four-domain pair and above but with the sacrifice of the computational efficiency. Figure 1(b) gives an example for inferring domain interactions from protein interaction and non-interaction data. It indicates that the classical probabilistic model fails to give the correct result for this case while our model can do it by considering multi-domain interactions.

### Selection of independent variables

In order to make the variables of model (1) independent with each other, we will delete dependent variables among *d*_*m*, *n*_, *d*_*mr*, *n *_and *d*_*r*, *n *_according to the following strategy. Define

Rm,n=Im,nNm,n,
 MathType@MTEF@5@5@+=feaafiart1ev1aaatCvAUfKttLearuWrP9MDH5MBPbIqV92AaeXatLxBI9gBaebbnrfifHhDYfgasaacH8akY=wiFfYdH8Gipec8Eeeu0xXdbba9frFj0=OqFfea0dXdd9vqai=hGuQ8kuc9pgc9s8qqaq=dirpe0xb9q8qiLsFr0=vr0=vr0dc8meaabaqaciaacaGaaeqabaqabeGadaaakeaacqWGsbGudaWgaaWcbaGaemyBa0MaeiilaWIaemOBa4gabeaakiabg2da9maalaaabaGaemysaK0aaSbaaSqaaiabd2gaTjabcYcaSiabd6gaUbqabaaakeaacqWGobGtdaWgaaWcbaGaemyBa0MaeiilaWIaemOBa4gabeaaaaGccqGGSaalaaa@3DA9@

where *I*_*mn *_is the number of interacting protein pairs in the training set that contain domain pair *D*_*m*, *n*_, and *N*_*m*, *n *_is the total number of protein pairs in the training set that contain *D*_*m*, *n*_. *R*_*mr*, *n *_and *R*_*r*, *n *_are similarly defined. For variables *d*_*m*, *n*_, *d*_*mr*, *n *_and *d*_*r*, *n*_, the variable deletion strategy is described by the following procedure.

1. If *R*_*mr*, *n*_*< R*_*m*, *n *_or *R*_*mr*, *n*_*< R*_*r*, *n*_, it indicates that the appearance frequency of domain pair *D*_*mr*, *n *_in interacting protein pairs is not higher than those of *D*_*m*, *n *_and *D*_*r*, *n*_. We consider that there is no cooperation between *D*_*m *_and *D*_*r *_in their interacting with *D*_*n*_, so we keep the variables *d*_*m*, *n*_, *d*_*r*, *n *_and delete the variable *d*_*mr*, *n *_in (1).

2. If *R*_*mr*, *n *_≥ *R*_*m*, *n *_and *R*_*mr*, *n *_≥ *R*_*r*, *n*_, for *D*_*m*, *n*_

• when *R*_*mr*, *n*_*> R*_*m*, *n *_and *I*_*mr*, *n *_= *I*_*m*, *n*_, the appearance frequency of domain pair *D*_*mr*, *n *_in interacting protein pairs is higher than those of *D*_*m*, *n *_and *D*_*r*, *n*_, and furthermore, *D*_*m*, *n *_does not appear in any other interacting protein pair without *D*_*r*_. Hence, we consider that *D*_*m *_and *D*_*r *_are cooperative when interacting with *D*_*n*_, and thereby the variable *d*_*m*, *n *_is deleted, but the variable *d*_*mr*, *n *_is kept in (1);

• when *R*_*mr*, *n *_= *R*_*m*, *n *_and *I*_*mr*, *n *_= *I*_*m*, *n*_, it means that *D*_*m *_and *D*_*r *_always appear together in individual proteins. Hence, *D*_*m *_and *D*_*r *_are considered as a superdomain and can be merged to one. For such a case, we delete variable *d*_*m*, *n *_but keep the cooperative-domain pair *d*_*mr*, *n*_.

The operations are performed in the same way for *D*_*r*, *n*_. For the case of Figure 1(b), variables for all domain pairs except (*D*_1 _- *D*_2_, *D*_3_) are deleted based on this procedure.

Obviously, the above operations do not cover the case *R*_*mr*, *n *_≥ *R*_*m*, *n *_and *I*_*mr*, *n*_*< I*_*mn*_. For this case, we cannot determine if or not there is a cooperative effect of domains *D*_*m *_and *D*_*r *_on their interacting with domain *D*_*n *_since *D*_*m*, *n *_also appears in the interacting pairs without *D*_*r*_, thereby we keep all of them. This may affect the assumption of independence, but there are few such cases. For example, for the date set MIPS1, among 22325 multi-domain pairs, there are only 85 such cases. Hence, by the above variable deletion operations, the assumption can be primarily satisfied. In the following formulation, all the variables appearing in the formula are those kept after the deleting strategy, whereas the probabilities of all deleted variables are set to be zero. Note that, in contrast to the appearance frequency or interaction strength for selecting cooperative domains, the two domains in a superdomain are determined based on their co-occurrence, and the cooperativity are also indirectly confirmed by their identical or similar functions from GO annotations.

### Inference of domain interactions

#### Linear programming with multi-domain pairs

Before predicting protein interactions, we need firstly to infer domain interactions from multiple datasets. Owing to experiment noises, each protein interaction dataset has a false positive rate *fp*^*k *^and a false negative rate *fn*^*k*^·*fp*^*k *^= Pr(oijk
 MathType@MTEF@5@5@+=feaafiart1ev1aaatCvAUfKttLearuWrP9MDH5MBPbIqV92AaeXatLxBI9gBaebbnrfifHhDYfgasaacH8akY=wiFfYdH8Gipec8Eeeu0xXdbba9frFj0=OqFfea0dXdd9vqai=hGuQ8kuc9pgc9s8qqaq=dirpe0xb9q8qiLsFr0=vr0=vr0dc8meaabaqaciaacaGaaeqabaqabeGadaaakeaacqWGVbWBdaqhaaWcbaGaemyAaKMaemOAaOgabaGaem4AaSgaaaaa@3257@ = 1|pijk
 MathType@MTEF@5@5@+=feaafiart1ev1aaatCvAUfKttLearuWrP9MDH5MBPbIqV92AaeXatLxBI9gBaebbnrfifHhDYfgasaacH8akY=wiFfYdH8Gipec8Eeeu0xXdbba9frFj0=OqFfea0dXdd9vqai=hGuQ8kuc9pgc9s8qqaq=dirpe0xb9q8qiLsFr0=vr0=vr0dc8meaabaqaciaacaGaaeqabaqabeGadaaakeaacqWGWbaCdaqhaaWcbaGaemyAaKMaemOAaOgabaGaem4AaSgaaaaa@3259@ = 0), *fn*^*k *^= Pr(oijk
 MathType@MTEF@5@5@+=feaafiart1ev1aaatCvAUfKttLearuWrP9MDH5MBPbIqV92AaeXatLxBI9gBaebbnrfifHhDYfgasaacH8akY=wiFfYdH8Gipec8Eeeu0xXdbba9frFj0=OqFfea0dXdd9vqai=hGuQ8kuc9pgc9s8qqaq=dirpe0xb9q8qiLsFr0=vr0=vr0dc8meaabaqaciaacaGaaeqabaqabeGadaaakeaacqWGVbWBdaqhaaWcbaGaemyAaKMaemOAaOgabaGaem4AaSgaaaaa@3257@ = 0|pijk
 MathType@MTEF@5@5@+=feaafiart1ev1aaatCvAUfKttLearuWrP9MDH5MBPbIqV92AaeXatLxBI9gBaebbnrfifHhDYfgasaacH8akY=wiFfYdH8Gipec8Eeeu0xXdbba9frFj0=OqFfea0dXdd9vqai=hGuQ8kuc9pgc9s8qqaq=dirpe0xb9q8qiLsFr0=vr0=vr0dc8meaabaqaciaacaGaaeqabaqabeGadaaakeaacqWGWbaCdaqhaaWcbaGaemyAaKMaemOAaOgabaGaem4AaSgaaaaa@3259@ = 1). where oijk
 MathType@MTEF@5@5@+=feaafiart1ev1aaatCvAUfKttLearuWrP9MDH5MBPbIqV92AaeXatLxBI9gBaebbnrfifHhDYfgasaacH8akY=wiFfYdH8Gipec8Eeeu0xXdbba9frFj0=OqFfea0dXdd9vqai=hGuQ8kuc9pgc9s8qqaq=dirpe0xb9q8qiLsFr0=vr0=vr0dc8meaabaqaciaacaGaaeqabaqabeGadaaakeaacqWGVbWBdaqhaaWcbaGaemyAaKMaemOAaOgabaGaem4AaSgaaaaa@3257@ = 1 if the interaction between proteins Pik
 MathType@MTEF@5@5@+=feaafiart1ev1aaatCvAUfKttLearuWrP9MDH5MBPbIqV92AaeXatLxBI9gBaebbnrfifHhDYfgasaacH8akY=wiFfYdH8Gipec8Eeeu0xXdbba9frFj0=OqFfea0dXdd9vqai=hGuQ8kuc9pgc9s8qqaq=dirpe0xb9q8qiLsFr0=vr0=vr0dc8meaabaqaciaacaGaaeqabaqabeGadaaakeaacqWGqbaudaqhaaWcbaGaemyAaKgabaGaem4AaSgaaaaa@30BC@ and Pjk
 MathType@MTEF@5@5@+=feaafiart1ev1aaatCvAUfKttLearuWrP9MDH5MBPbIqV92AaeXatLxBI9gBaebbnrfifHhDYfgasaacH8akY=wiFfYdH8Gipec8Eeeu0xXdbba9frFj0=OqFfea0dXdd9vqai=hGuQ8kuc9pgc9s8qqaq=dirpe0xb9q8qiLsFr0=vr0=vr0dc8meaabaqaciaacaGaaeqabaqabeGadaaakeaacqWGqbaudaqhaaWcbaGaemOAaOgabaGaem4AaSgaaaaa@30BE@ is observed in the dataset and oijk
 MathType@MTEF@5@5@+=feaafiart1ev1aaatCvAUfKttLearuWrP9MDH5MBPbIqV92AaeXatLxBI9gBaebbnrfifHhDYfgasaacH8akY=wiFfYdH8Gipec8Eeeu0xXdbba9frFj0=OqFfea0dXdd9vqai=hGuQ8kuc9pgc9s8qqaq=dirpe0xb9q8qiLsFr0=vr0=vr0dc8meaabaqaciaacaGaaeqabaqabeGadaaakeaacqWGVbWBdaqhaaWcbaGaemyAaKMaemOAaOgabaGaem4AaSgaaaaa@3257@ = 0 otherwise. Thus the probability that proteins Pik
 MathType@MTEF@5@5@+=feaafiart1ev1aaatCvAUfKttLearuWrP9MDH5MBPbIqV92AaeXatLxBI9gBaebbnrfifHhDYfgasaacH8akY=wiFfYdH8Gipec8Eeeu0xXdbba9frFj0=OqFfea0dXdd9vqai=hGuQ8kuc9pgc9s8qqaq=dirpe0xb9q8qiLsFr0=vr0=vr0dc8meaabaqaciaacaGaaeqabaqabeGadaaakeaacqWGqbaudaqhaaWcbaGaemyAaKgabaGaem4AaSgaaaaa@30BC@ and Pjk
 MathType@MTEF@5@5@+=feaafiart1ev1aaatCvAUfKttLearuWrP9MDH5MBPbIqV92AaeXatLxBI9gBaebbnrfifHhDYfgasaacH8akY=wiFfYdH8Gipec8Eeeu0xXdbba9frFj0=OqFfea0dXdd9vqai=hGuQ8kuc9pgc9s8qqaq=dirpe0xb9q8qiLsFr0=vr0=vr0dc8meaabaqaciaacaGaaeqabaqabeGadaaakeaacqWGqbaudaqhaaWcbaGaemOAaOgabaGaem4AaSgaaaaa@30BE@ in dataset *k *are observed to be interacting in the experiments is related with the real interaction probability in the following way:

Pr⁡(oijk=1)=Pr⁡(pijk=1)(1−fnk)+(1−Pr⁡(pijk=1))fpk.
 MathType@MTEF@5@5@+=feaafiart1ev1aaatCvAUfKttLearuWrP9MDH5MBPbIqV92AaeXatLxBI9gBaebbnrfifHhDYfgasaacH8akY=wiFfYdH8Gipec8Eeeu0xXdbba9frFj0=OqFfea0dXdd9vqai=hGuQ8kuc9pgc9s8qqaq=dirpe0xb9q8qiLsFr0=vr0=vr0dc8meaabaqaciaacaGaaeqabaqabeGadaaakeaacyGGqbaucqGGYbGCcqGGOaakcqWGVbWBdaqhaaWcbaGaemyAaKMaemOAaOgabaGaem4AaSgaaOGaeyypa0JaeGymaeJaeiykaKIaeyypa0JagiiuaaLaeiOCaiNaeiikaGIaemiCaa3aa0baaSqaaiabdMgaPjabdQgaQbqaaiabdUgaRbaakiabg2da9iabigdaXiabcMcaPiabcIcaOiabigdaXiabgkHiTiabdAgaMjabd6gaUnaaCaaaleqabaGaem4AaSgaaOGaeiykaKIaey4kaSIaeiikaGIaeGymaeJaeyOeI0IagiiuaaLaeiOCaiNaeiikaGIaemiCaa3aa0baaSqaaiabdMgaPjabdQgaQbqaaiabdUgaRbaakiabg2da9iabigdaXiabcMcaPiabcMcaPiabdAgaMjabdchaWnaaCaaaleqabaGaem4AaSgaaOGaeiOla4caaa@6317@

The parameters *fp*^*k *^and *fn*^*k *^can be estimated from experimental data in a similar way as that in Liu et al. [14].

With the basic probabilistic model (1) and the formula (3), we have

Pr⁡(oijk=1)−fpk1−fnk−fpk=1−∏Dm,n∈Pijk(1−Pr⁡(dm,n=1))∏Dmr,n∈Pijk(1−Pr⁡(dmr,n=1))∏Dm,nr∈Pijk(1−Pr⁡(dm,nr=1))
 MathType@MTEF@5@5@+=feaafiart1ev1aaatCvAUfKttLearuWrP9MDH5MBPbIqV92AaeXatLxBI9gBaebbnrfifHhDYfgasaacH8akY=wiFfYdH8Gipec8Eeeu0xXdbba9frFj0=OqFfea0dXdd9vqai=hGuQ8kuc9pgc9s8qqaq=dirpe0xb9q8qiLsFr0=vr0=vr0dc8meaabaqaciaacaGaaeqabaqabeGadaaakeaadaWcaaqaaiGbccfaqjabckhaYjabcIcaOiabd+gaVnaaDaaaleaacqWGPbqAcqWGQbGAaeaacqWGRbWAaaGccqGH9aqpcqaIXaqmcqGGPaqkcqGHsislcqWGMbGzcqWGWbaCdaahaaWcbeqaaiabdUgaRbaaaOqaaiabigdaXiabgkHiTiabdAgaMjabd6gaUnaaCaaaleqabaGaem4AaSgaaOGaeyOeI0IaemOzayMaemiCaa3aaWbaaSqabeaacqWGRbWAaaaaaOGaeyypa0JaeGymaeJaeyOeI0YaaebuaeaacqGGOaakcqaIXaqmcqGHsislcyGGqbaucqGGYbGCcqGGOaakcqWGKbazdaWgaaWcbaGaemyBa0MaeiilaWIaemOBa4gabeaakiabg2da9iabigdaXiabcMcaPiabcMcaPaWcbaGaemiraq0aaSbaaWqaaiabd2gaTjabcYcaSiabd6gaUbqabaWccqGHiiIZcqWGqbaudaqhaaadbaGaemyAaKMaemOAaOgabaGaem4AaSgaaaWcbeqdcqGHpis1aOWaaebuaeaacqGGOaakcqaIXaqmcqGHsislcyGGqbaucqGGYbGCcqGGOaakcqWGKbazdaWgaaWcbaGaemyBa0MaemOCaiNaeiilaWIaemOBa4gabeaakiabg2da9iabigdaXiabcMcaPiabcMcaPaWcbaGaemiraq0aaSbaaWqaaiabd2gaTjabdkhaYjabcYcaSiabd6gaUbqabaWccqGHiiIZcqWGqbaudaqhaaadbaGaemyAaKMaemOAaOgabaGaem4AaSgaaaWcbeqdcqGHpis1aOWaaebuaeaacqGGOaakcqaIXaqmcqGHsislcyGGqbaucqGGYbGCcqGGOaakcqWGKbazdaWgaaWcbaGaemyBa0MaeiilaWIaemOBa4MaemOCaihabeaakiabg2da9iabigdaXiabcMcaPiabcMcaPaWcbaGaemiraq0aaSbaaWqaaiabd2gaTjabcYcaSiabd6gaUjabdkhaYbqabaWccqGHiiIZcqWGqbaudaqhaaadbaGaemyAaKMaemOAaOgabaGaem4AaSgaaaWcbeqdcqGHpis1aaaa@A8FD@

Pr(oijk
 MathType@MTEF@5@5@+=feaafiart1ev1aaatCvAUfKttLearuWrP9MDH5MBPbIqV92AaeXatLxBI9gBaebbnrfifHhDYfgasaacH8akY=wiFfYdH8Gipec8Eeeu0xXdbba9frFj0=OqFfea0dXdd9vqai=hGuQ8kuc9pgc9s8qqaq=dirpe0xb9q8qiLsFr0=vr0=vr0dc8meaabaqaciaacaGaaeqabaqabeGadaaakeaacqWGVbWBdaqhaaWcbaGaemyAaKMaemOAaOgabaGaem4AaSgaaaaa@3257@ = 1) when two proteins (Pik
 MathType@MTEF@5@5@+=feaafiart1ev1aaatCvAUfKttLearuWrP9MDH5MBPbIqV92AaeXatLxBI9gBaebbnrfifHhDYfgasaacH8akY=wiFfYdH8Gipec8Eeeu0xXdbba9frFj0=OqFfea0dXdd9vqai=hGuQ8kuc9pgc9s8qqaq=dirpe0xb9q8qiLsFr0=vr0=vr0dc8meaabaqaciaacaGaaeqabaqabeGadaaakeaacqWGqbaudaqhaaWcbaGaemyAaKgabaGaem4AaSgaaaaa@30BC@, Pjk
 MathType@MTEF@5@5@+=feaafiart1ev1aaatCvAUfKttLearuWrP9MDH5MBPbIqV92AaeXatLxBI9gBaebbnrfifHhDYfgasaacH8akY=wiFfYdH8Gipec8Eeeu0xXdbba9frFj0=OqFfea0dXdd9vqai=hGuQ8kuc9pgc9s8qqaq=dirpe0xb9q8qiLsFr0=vr0=vr0dc8meaabaqaciaacaGaaeqabaqabeGadaaakeaacqWGqbaudaqhaaWcbaGaemOAaOgabaGaem4AaSgaaaaa@30BE@) interact and 0 otherwise in the binary PPI data. For numerical interaction data we can set Pr(oijk
 MathType@MTEF@5@5@+=feaafiart1ev1aaatCvAUfKttLearuWrP9MDH5MBPbIqV92AaeXatLxBI9gBaebbnrfifHhDYfgasaacH8akY=wiFfYdH8Gipec8Eeeu0xXdbba9frFj0=OqFfea0dXdd9vqai=hGuQ8kuc9pgc9s8qqaq=dirpe0xb9q8qiLsFr0=vr0=vr0dc8meaabaqaciaacaGaaeqabaqabeGadaaakeaacqWGVbWBdaqhaaWcbaGaemyAaKMaemOAaOgabaGaem4AaSgaaaaa@3257@ = 1)as the ratio of interactions between proteins Pik
 MathType@MTEF@5@5@+=feaafiart1ev1aaatCvAUfKttLearuWrP9MDH5MBPbIqV92AaeXatLxBI9gBaebbnrfifHhDYfgasaacH8akY=wiFfYdH8Gipec8Eeeu0xXdbba9frFj0=OqFfea0dXdd9vqai=hGuQ8kuc9pgc9s8qqaq=dirpe0xb9q8qiLsFr0=vr0=vr0dc8meaabaqaciaacaGaaeqabaqabeGadaaakeaacqWGqbaudaqhaaWcbaGaemyAaKgabaGaem4AaSgaaaaa@30BC@ and Pjk
 MathType@MTEF@5@5@+=feaafiart1ev1aaatCvAUfKttLearuWrP9MDH5MBPbIqV92AaeXatLxBI9gBaebbnrfifHhDYfgasaacH8akY=wiFfYdH8Gipec8Eeeu0xXdbba9frFj0=OqFfea0dXdd9vqai=hGuQ8kuc9pgc9s8qqaq=dirpe0xb9q8qiLsFr0=vr0=vr0dc8meaabaqaciaacaGaaeqabaqabeGadaaakeaacqWGqbaudaqhaaWcbaGaemOAaOgabaGaem4AaSgaaaaa@30BE@ in a series of experiments. Note that the left side may be greater than 1 due to the incomplete interaction information in binary experiment data. For such a case we can normalize them first. Let *x*_*m*, *n *_= ln(1- Pr(*d*_*m*, *n *_= 1)), *x*_*mr*, *n *_= ln(1 - Pr(*d*_*mr*, *n *_= 1)), *x*_*m*, *nr *_= ln(1 - Pr(*d*_*m*, *nr *_= 1)) and βijk=ln⁡(1−Pr⁡(oijk=1)−fpk1−fnk−fpk)
 MathType@MTEF@5@5@+=feaafiart1ev1aaatCvAUfKttLearuWrP9MDH5MBPbIqV92AaeXatLxBI9gBaebbnrfifHhDYfgasaacH8akY=wiFfYdH8Gipec8Eeeu0xXdbba9frFj0=OqFfea0dXdd9vqai=hGuQ8kuc9pgc9s8qqaq=dirpe0xb9q8qiLsFr0=vr0=vr0dc8meaabaqaciaacaGaaeqabaqabeGadaaakeaaiiGacqWFYoGydaqhaaWcbaGaemyAaKMaemOAaOgabaGaem4AaSgaaOGaeyypa0JagiiBaWMaeiOBa4MaeiikaGIaeGymaeJaeyOeI0YaaSaaaeaacyGGqbaucqGGYbGCcqGGOaakcqWGVbWBdaqhaaWcbaGaemyAaKMaemOAaOgabaGaem4AaSgaaOGaeyypa0JaeGymaeJaeiykaKIaeyOeI0IaemOzayMaemiCaa3aaWbaaSqabeaacqWGRbWAaaaakeaacqaIXaqmcqGHsislcqWGMbGzcqWGUbGBdaahaaWcbeqaaiabdUgaRbaakiabgkHiTiabdAgaMjabdchaWnaaCaaaleqabaGaem4AaSgaaaaakiabcMcaPaaa@56AF@. By the similar technique adopted in Hayashida et al. [16], then the above equalities can be written as

∑Dm,n∈Pijkxm,n+∑Dmr,n∈Pijkxmr,n+∑Dm,nr∈Pijkxm,nr=βijk.
 MathType@MTEF@5@5@+=feaafiart1ev1aaatCvAUfKttLearuWrP9MDH5MBPbIqV92AaeXatLxBI9gBaebbnrfifHhDYfgasaacH8akY=wiFfYdH8Gipec8Eeeu0xXdbba9frFj0=OqFfea0dXdd9vqai=hGuQ8kuc9pgc9s8qqaq=dirpe0xb9q8qiLsFr0=vr0=vr0dc8meaabaqaciaacaGaaeqabaqabeGadaaakeaadaaeqbqaaiabdIha4naaBaaaleaacqWGTbqBcqGGSaalcqWGUbGBaeqaaaqaaiabdseaenaaBaaameaacqWGTbqBcqGGSaalcqWGUbGBaeqaaSGaeyicI4Saemiuaa1aa0baaWqaaiabdMgaPjabdQgaQbqaaiabdUgaRbaaaSqab0GaeyyeIuoakiabgUcaRmaaqafabaGaemiEaG3aaSbaaSqaaiabd2gaTjabdkhaYjabcYcaSiabd6gaUbqabaaabaGaemiraq0aaSbaaWqaaiabd2gaTjabdkhaYjabcYcaSiabd6gaUbqabaWccqGHiiIZcqWGqbaudaqhaaadbaGaemyAaKMaemOAaOgabaGaem4AaSgaaaWcbeqdcqGHris5aOGaey4kaSYaaabuaeaacqWG4baEdaWgaaWcbaGaemyBa0MaeiilaWIaemOBa4MaemOCaihabeaakiabg2da9GGaciab=j7aInaaDaaaleaacqWGPbqAcqWGQbGAaeaacqWGRbWAaaaabaGaemiraq0aaSbaaWqaaiabd2gaTjabcYcaSiabd6gaUjabdkhaYbqabaWccqGHiiIZcqWGqbaudaqhaaadbaGaemyAaKMaemOAaOgabaGaem4AaSgaaaWcbeqdcqGHris5aOGaeiOla4caaa@761B@

This is a set of linear equalities. If we can find *x*_*m*, *n*_, *x*_*mr*, *n *_and *x*_*m*, *nr *_(*x*_*m*, *n *_≤ 0, *x*_*mr*, *n *_≤ 0 and *x*_*m*, *nr *_≤ 0) satisfying (5) for all observed protein interaction data, the domain interaction probabilities Pr(*d*_*m*, *n *_= 1), Pr(*d*_*mr*, *n *_= 1) and Pr(*d*_*m*, *nr *_= 1) fully consistent with the training data can be obtained. However, it is usually impossible to satisfy all constraints owing to the noise and incompleteness of experimental data. In such a case it is natural and reasonable to minimize the total error with respect to *L*_1 _norm. Therefore we can obtain the following Linear Programming with Multi-domain pairs (LPM):

min⁡ε, x∑Pijk|εijk|s.t.∑Dm,n∈Pijkxm,n+∑Dmr,n∈Pijkxmr,n+∑Dm,nr∈Pijkxm,nr=βijk−εijk for all Pijk,xm,n≤0,xmr,n≤0,xm,nr≤0,i,j∈{1,...,Nk},k=1,⋯,K
 MathType@MTEF@5@5@+=feaafiart1ev1aaatCvAUfKttLearuWrP9MDH5MBPbIqV92AaeXatLxBI9gBaebbnrfifHhDYfgasaacH8akY=wiFfYdH8Gipec8Eeeu0xXdbba9frFj0=OqFfea0dXdd9vqai=hGuQ8kuc9pgc9s8qqaq=dirpe0xb9q8qiLsFr0=vr0=vr0dc8meaabaqaciaacaGaaeqabaqabeGadaaakeaafaqaaeabcaaaaeaadaWfqaqaaiGbc2gaTjabcMgaPjabc6gaUbWcbaacciGae8xTduMaeiilaWIaeeiiaaIaemiEaGhabeaaaOqaamaaqafabaWaaqWaaeaacqWF1oqzdaqhaaWcbaGaemyAaKMaemOAaOgabaGaem4AaSgaaaGccaGLhWUaayjcSdaaleaacqWGqbaudaqhaaadbaGaemyAaKMaemOAaOgabaGaem4AaSgaaaWcbeqdcqGHris5aaGcbaGaem4CamNaeiOla4IaemiDaqNaeiOla4cabaWaaabuaeaacqWG4baEdaWgaaWcbaGaemyBa0MaeiilaWIaemOBa4gabeaaaeaacqWGebardaWgaaadbaGaemyBa0MaeiilaWIaemOBa4gabeaaliabgIGiolabdcfaqnaaDaaameaacqWGPbqAcqWGQbGAaeaacqWGRbWAaaaaleqaniabggHiLdGccqGHRaWkdaaeqbqaaiabdIha4naaBaaaleaacqWGTbqBcqWGYbGCcqGGSaalcqWGUbGBaeqaaaqaaiabdseaenaaBaaameaacqWGTbqBcqWGYbGCcqGGSaalcqWGUbGBaeqaaSGaeyicI4Saemiuaa1aa0baaWqaaiabdMgaPjabdQgaQbqaaiabdUgaRbaaaSqab0GaeyyeIuoakiabgUcaRmaaqafabaGaemiEaG3aaSbaaSqaaiabd2gaTjabcYcaSiabd6gaUjabdkhaYbqabaGccqGH9aqpcqWFYoGydaqhaaWcbaGaemyAaKMaemOAaOgabaGaem4AaSgaaOGaeyOeI0Iae8xTdu2aa0baaSqaaiabdMgaPjabdQgaQbqaaiabdUgaRbaakiabbccaGiabbAgaMjabb+gaVjabbkhaYjabbccaGiabbggaHjabbYgaSjabbYgaSjabbccaGiabdcfaqnaaDaaaleaacqWGPbqAcqWGQbGAaeaacqWGRbWAaaGccqGGSaalaSqaaiabdseaenaaBaaameaacqWGTbqBcqGGSaalcqWGUbGBcqWGYbGCaeqaaSGaeyicI4Saemiuaa1aa0baaWqaaiabdMgaPjabdQgaQbqaaiabdUgaRbaaaSqab0GaeyyeIuoaaOqaaaqaaiabdIha4naaBaaaleaacqWGTbqBcqGGSaalcqWGUbGBaeqaaOGaeyizImQaeGimaaJaeiilaWIaemiEaG3aaSbaaSqaaiabd2gaTjabdkhaYjabcYcaSiabd6gaUbqabaGccqGHKjYOcqaIWaamcqGGSaalcqWG4baEdaWgaaWcbaGaemyBa0MaeiilaWIaemOBa4MaemOCaihabeaakiabgsMiJkabicdaWiabcYcaSaqaaaqaauaabeqabiaaaeaacqWGPbqAcqGGSaalcqWGQbGAcqGHiiIZcqGG7bWEcqaIXaqmcqGGSaalcqGGUaGlcqGGUaGlcqGGUaGlcqGGSaalcqWGobGtdaWgaaWcbaGaem4AaSgabeaakiabc2ha9jabcYcaSaqaaiabdUgaRjabg2da9iabigdaXiabcYcaSiabl+UimjabcYcaSiabdUealbaaaaaaaa@E244@

where εi,jk
 MathType@MTEF@5@5@+=feaafiart1ev1aaatCvAUfKttLearuWrP9MDH5MBPbIqV92AaeXatLxBI9gBaebbnrfifHhDYfgasaacH8akY=wiFfYdH8Gipec8Eeeu0xXdbba9frFj0=OqFfea0dXdd9vqai=hGuQ8kuc9pgc9s8qqaq=dirpe0xb9q8qiLsFr0=vr0=vr0dc8meaabaqaciaacaGaaeqabaqabeGadaaakeaaiiGacqWF1oqzdaqhaaWcbaGaemyAaKMaeiilaWIaemOAaOgabaGaem4AaSgaaaaa@337E@ is the error for each equality of (5). Model (6) can be solved by any standard LP technique (see Additional file 1).

Solving (6), we can obtain a set of interaction probabilities for domain pairs. Then, new protein interactions can be predicted by these inferred domain interactions through the probabilistic model (1).

#### Association probabilistic method with multi-domain pairs

Numerical experiments show that LPM performs well, but is computationally expensive for large scale problems. Therefore, we introduce a faster probabilistic method based on statistics. This method is based on a generalization of Association Probabilistic Method [13] with multi-domain pairs (APMM). It estimates the interaction probabilities of multi-domain pairs in the following way:

Pr⁡(dm,n=1)=∑{Pijk|Dm,n∈Pijk}[1−(1−ρijk)|Pijk|]Nm,n,Pr⁡(dmr,n=1)=∑{Pijk|Dmr,n∈Pijk}[1−(1−ρijk)|Pijk|]Nmr,n,Pr⁡(dm,nr=1)=∑{Pijk|Dm,nr∈Pijk}[1−(1−ρijk)|Pijk|]Nm,nr,
 MathType@MTEF@5@5@+=feaafiart1ev1aaatCvAUfKttLearuWrP9MDH5MBPbIqV92AaeXatLxBI9gBaebbnrfifHhDYfgasaacH8akY=wiFfYdH8Gipec8Eeeu0xXdbba9frFj0=OqFfea0dXdd9vqai=hGuQ8kuc9pgc9s8qqaq=dirpe0xb9q8qiLsFr0=vr0=vr0dc8meaabaqaciaacaGaaeqabaqabeGadaaakeaafaqabeWabaaabaGagiiuaaLaeiOCaiNaeiikaGIaemizaq2aaSbaaSqaaiabd2gaTjabcYcaSiabd6gaUbqabaGccqGH9aqpcqaIXaqmcqGGPaqkcqGH9aqpdaWcaaqaamaaqababaGaei4waSLaeGymaeJaeyOeI0IaeiikaGIaeGymaeJaeyOeI0ccciGae8xWdi3aa0baaSqaaiabdMgaPjabdQgaQbqaaiabdUgaRbaakiabcMcaPmaaCaaaleqabaGaeiiFaWNaemiuaa1aa0baaWqaaiabdMgaPjabdQgaQbqaaiabdUgaRbaaliabcYha8baakiabc2faDbWcbaGaei4EaSNaemiuaa1aa0baaWqaaiabdMgaPjabdQgaQbqaaiabdUgaRbaaliabcYha8jabdseaenaaBaaameaacqWGTbqBcqGGSaalcqWGUbGBaeqaaSGaeyicI4Saemiuaa1aa0baaWqaaiabdMgaPjabdQgaQbqaaiabdUgaRbaaliabc2ha9bqab0GaeyyeIuoaaOqaaiabd6eaonaaBaaaleaacqWGTbqBcqGGSaalcqWGUbGBaeqaaaaakiabcYcaSaqaaiGbccfaqjabckhaYjabcIcaOiabdsgaKnaaBaaaleaacqWGTbqBcqWGYbGCcqGGSaalcqWGUbGBaeqaaOGaeyypa0JaeGymaeJaeiykaKIaeyypa0ZaaSaaaeaadaaeqaqaaiabcUfaBjabigdaXiabgkHiTiabcIcaOiabigdaXiabgkHiTiab=f8aYnaaDaaaleaacqWGPbqAcqWGQbGAaeaacqWGRbWAaaGccqGGPaqkdaahaaWcbeqaaiabcYha8jabdcfaqnaaDaaameaacqWGPbqAcqWGQbGAaeaacqWGRbWAaaWccqGG8baFaaGccqGGDbqxaSqaaiabcUha7jabdcfaqnaaDaaameaacqWGPbqAcqWGQbGAaeaacqWGRbWAaaWccqGG8baFcqWGebardaWgaaadbaGaemyBa0MaemOCaiNaeiilaWIaemOBa4gabeaaliabgIGiolabdcfaqnaaDaaameaacqWGPbqAcqWGQbGAaeaacqWGRbWAaaWccqGG9bqFaeqaniabggHiLdaakeaacqWGobGtdaWgaaWcbaGaemyBa0MaemOCaiNaeiilaWIaemOBa4gabeaaaaGccqGGSaalaeaacyGGqbaucqGGYbGCcqGGOaakcqWGKbazdaWgaaWcbaGaemyBa0MaeiilaWIaemOBa4MaemOCaihabeaakiabg2da9iabigdaXiabcMcaPiabg2da9maalaaabaWaaabeaeaacqGGBbWwcqaIXaqmcqGHsislcqGGOaakcqaIXaqmcqGHsislcqWFbpGCdaqhaaWcbaGaemyAaKMaemOAaOgabaGaem4AaSgaaOGaeiykaKYaaWbaaSqabeaacqGG8baFcqWGqbaudaqhaaadbaGaemyAaKMaemOAaOgabaGaem4AaSgaaSGaeiiFaWhaaOGaeiyxa0faleaacqGG7bWEcqWGqbaudaqhaaadbaGaemyAaKMaemOAaOgabaGaem4AaSgaaSGaeiiFaWNaemiraq0aaSbaaWqaaiabd2gaTjabcYcaSiabd6gaUjabdkhaYbqabaWccqGHiiIZcqWGqbaudaqhaaadbaGaemyAaKMaemOAaOgabaGaem4AaSgaaSGaeiyFa0habeqdcqGHris5aaGcbaGaemOta40aaSbaaSqaaiabd2gaTjabcYcaSiabd6gaUjabdkhaYbqabaaaaOGaeiilaWcaaaaa@F7F2@

where |Pijk
 MathType@MTEF@5@5@+=feaafiart1ev1aaatCvAUfKttLearuWrP9MDH5MBPbIqV92AaeXatLxBI9gBaebbnrfifHhDYfgasaacH8akY=wiFfYdH8Gipec8Eeeu0xXdbba9frFj0=OqFfea0dXdd9vqai=hGuQ8kuc9pgc9s8qqaq=dirpe0xb9q8qiLsFr0=vr0=vr0dc8meaabaqaciaacaGaaeqabaqabeGadaaakeaacqWGqbaudaqhaaWcbaGaemyAaKMaemOAaOgabaGaem4AaSgaaaaa@3219@| represents the number of multi-domain pairs in Pijk
 MathType@MTEF@5@5@+=feaafiart1ev1aaatCvAUfKttLearuWrP9MDH5MBPbIqV92AaeXatLxBI9gBaebbnrfifHhDYfgasaacH8akY=wiFfYdH8Gipec8Eeeu0xXdbba9frFj0=OqFfea0dXdd9vqai=hGuQ8kuc9pgc9s8qqaq=dirpe0xb9q8qiLsFr0=vr0=vr0dc8meaabaqaciaacaGaaeqabaqabeGadaaakeaacqWGqbaudaqhaaWcbaGaemyAaKMaemOAaOgabaGaem4AaSgaaaaa@3219@, and ρijk
 MathType@MTEF@5@5@+=feaafiart1ev1aaatCvAUfKttLearuWrP9MDH5MBPbIqV92AaeXatLxBI9gBaebbnrfifHhDYfgasaacH8akY=wiFfYdH8Gipec8Eeeu0xXdbba9frFj0=OqFfea0dXdd9vqai=hGuQ8kuc9pgc9s8qqaq=dirpe0xb9q8qiLsFr0=vr0=vr0dc8meaabaqaciaacaGaaeqabaqabeGadaaakeaaiiGacqWFbpGCdaqhaaWcbaGaemyAaKMaemOAaOgabaGaem4AaSgaaaaa@32B7@ is the observed interaction probability between Pik
 MathType@MTEF@5@5@+=feaafiart1ev1aaatCvAUfKttLearuWrP9MDH5MBPbIqV92AaeXatLxBI9gBaebbnrfifHhDYfgasaacH8akY=wiFfYdH8Gipec8Eeeu0xXdbba9frFj0=OqFfea0dXdd9vqai=hGuQ8kuc9pgc9s8qqaq=dirpe0xb9q8qiLsFr0=vr0=vr0dc8meaabaqaciaacaGaaeqabaqabeGadaaakeaacqWGqbaudaqhaaWcbaGaemyAaKgabaGaem4AaSgaaaaa@30BC@ and Pjk
 MathType@MTEF@5@5@+=feaafiart1ev1aaatCvAUfKttLearuWrP9MDH5MBPbIqV92AaeXatLxBI9gBaebbnrfifHhDYfgasaacH8akY=wiFfYdH8Gipec8Eeeu0xXdbba9frFj0=OqFfea0dXdd9vqai=hGuQ8kuc9pgc9s8qqaq=dirpe0xb9q8qiLsFr0=vr0=vr0dc8meaabaqaciaacaGaaeqabaqabeGadaaakeaacqWGqbaudaqhaaWcbaGaemOAaOgabaGaem4AaSgaaaaa@30BE@ in the experimental data after considering the false positive and false negative rates. Note that the deleted variables are not counted. From these formula, we can see that all domain pairs have an equal opportunity to contribute the interactions between Pik
 MathType@MTEF@5@5@+=feaafiart1ev1aaatCvAUfKttLearuWrP9MDH5MBPbIqV92AaeXatLxBI9gBaebbnrfifHhDYfgasaacH8akY=wiFfYdH8Gipec8Eeeu0xXdbba9frFj0=OqFfea0dXdd9vqai=hGuQ8kuc9pgc9s8qqaq=dirpe0xb9q8qiLsFr0=vr0=vr0dc8meaabaqaciaacaGaaeqabaqabeGadaaakeaacqWGqbaudaqhaaWcbaGaemyAaKgabaGaem4AaSgaaaaa@30BC@ and Pjk
 MathType@MTEF@5@5@+=feaafiart1ev1aaatCvAUfKttLearuWrP9MDH5MBPbIqV92AaeXatLxBI9gBaebbnrfifHhDYfgasaacH8akY=wiFfYdH8Gipec8Eeeu0xXdbba9frFj0=OqFfea0dXdd9vqai=hGuQ8kuc9pgc9s8qqaq=dirpe0xb9q8qiLsFr0=vr0=vr0dc8meaabaqaciaacaGaaeqabaqabeGadaaakeaacqWGqbaudaqhaaWcbaGaemOAaOgabaGaem4AaSgaaaaa@30BE@ for |Pijk
 MathType@MTEF@5@5@+=feaafiart1ev1aaatCvAUfKttLearuWrP9MDH5MBPbIqV92AaeXatLxBI9gBaebbnrfifHhDYfgasaacH8akY=wiFfYdH8Gipec8Eeeu0xXdbba9frFj0=OqFfea0dXdd9vqai=hGuQ8kuc9pgc9s8qqaq=dirpe0xb9q8qiLsFr0=vr0=vr0dc8meaabaqaciaacaGaaeqabaqabeGadaaakeaacqWGqbaudaqhaaWcbaGaemyAaKMaemOAaOgabaGaem4AaSgaaaaa@3219@| > 1 under the independence assumption for domain interactions. With these interaction probabilities of domain pairs, we can predict whether a pair of proteins interact or not by the formula (1). The computation of this method is much simple and thus highly efficient. In addition, it does not require any parameter tuning.

### Evaluation measures

We validated our method using several types of experiments with different criteria. For computing the similarity of GO annotations [40], we adopted a simple method used successfully in Chen et al. [41] and Wu et al. [42]. In this method, known proteins are assigned with functional annotations by a GO Identification (ID). According to the hierarchical structure of GO annotations, each GO term corresponds to a numerical GO INDEX. The more detailed level of the GO INDEX, the more specific is the function assigned to a protein. The maximum level of GO INDEX is 14. The function similarity between proteins *P*_*x *_and *P*_*y *_is defined by the maximum number of index levels from the top shared by *P*_*x *_and *P*_*y*_. The smaller the value of function similarity, the broader is the functional category shared by the two proteins. The details can be found in Chen et al. [41].

For protein interaction prediction on numerical PPI data, we use root-mean-square error (RMSE) to measure the difference between the observed probability values and the predicted probability values:

RMSE=∑Pijk∈P(Pr⁡(Pijk)=1)−ρijk)/|P|
 MathType@MTEF@5@5@+=feaafiart1ev1aaatCvAUfKttLearuWrP9MDH5MBPbIqV92AaeXatLxBI9gBaebbnrfifHhDYfgasaacH8akY=wiFfYdH8Gipec8Eeeu0xXdbba9frFj0=OqFfea0dXdd9vqai=hGuQ8kuc9pgc9s8qqaq=dirpe0xb9q8qiLsFr0=vr0=vr0dc8meaabaqaciaacaGaaeqabaqabeGadaaakeaacqqGsbGucqqGnbqtcqqGtbWucqqGfbqrcqGH9aqpdaGcaaqaamaaqafabaGaeiikaGIagiiuaaLaeiOCaiNaeiikaGIaemiuaa1aa0baaSqaaiabdMgaPjabdQgaQbqaaiabdUgaRbaakiabcMcaPiabg2da9iabigdaXiabcMcaPiabgkHiTGGaciab=f8aYnaaDaaaleaacqWGPbqAcqWGQbGAaeaacqWGRbWAaaGccqGGPaqkcqGGVaWldaabdaqaaiabdcfaqbGaay5bSlaawIa7aaWcbaGaemiuaa1aa0baaWqaaiabdMgaPjabdQgaQbqaaiabdUgaRbaaliabgIGiolabdcfaqbqab0GaeyyeIuoaaSqabaaaaa@5714@

where *P *denotes a set of protein pairs (training set or testing set) including interactions and non-interactions. Non-interacting protein pairs may be those not appearing in the observed interaction data or those whose interaction probabilities are below a threshold.

For protein interaction prediction on binary PPI data, we use sensitivity and specificity to evaluate the performance of a method. Specifically, given a set of interacting protein pairs as positive set and a non-interacting protein pair set as negative set, sensitivity and specificity (denoted by SN and SP) are respectively defined as

SN=TPTP+FN,
 MathType@MTEF@5@5@+=feaafiart1ev1aaatCvAUfKttLearuWrP9MDH5MBPbIqV92AaeXatLxBI9gBaebbnrfifHhDYfgasaacH8akY=wiFfYdH8Gipec8Eeeu0xXdbba9frFj0=OqFfea0dXdd9vqai=hGuQ8kuc9pgc9s8qqaq=dirpe0xb9q8qiLsFr0=vr0=vr0dc8meaabaqaciaacaGaaeqabaqabeGadaaakeaacqqGtbWucqqGobGtcqGH9aqpdaWcaaqaaiabbsfaujabbcfaqbqaaiabbsfaujabbcfaqjabgUcaRiabbAeagjabb6eaobaacqGGSaalaaa@38B6@

SP=TNTN+FP,
 MathType@MTEF@5@5@+=feaafiart1ev1aaatCvAUfKttLearuWrP9MDH5MBPbIqV92AaeXatLxBI9gBaebbnrfifHhDYfgasaacH8akY=wiFfYdH8Gipec8Eeeu0xXdbba9frFj0=OqFfea0dXdd9vqai=hGuQ8kuc9pgc9s8qqaq=dirpe0xb9q8qiLsFr0=vr0=vr0dc8meaabaqaciaacaGaaeqabaqabeGadaaakeaacqqGtbWucqqGqbaucqGH9aqpdaWcaaqaaiabbsfaujabb6eaobqaaiabbsfaujabb6eaojabgUcaRiabbAeagjabbcfaqbaacqGGSaalaaa@38B6@

where the number of true positives (TP), true negatives (TN), false positives (FP) and false negatives (FN) are estimated with respect to the given test set.

The evaluation of the predicted domain interactions in this work is based on the overlap with the gold standard set iPfam. We adopted binomial cumulative distribution function to compute the significance of the overlap (*p*-value) by comparing with randomly predicted domain pairs:

P=1−∑k=0N(nk)pk(1−p)n−k
 MathType@MTEF@5@5@+=feaafiart1ev1aaatCvAUfKttLearuWrP9MDH5MBPbIqV92AaeXatLxBI9gBaebbnrfifHhDYfgasaacH8akY=wiFfYdH8Gipec8Eeeu0xXdbba9frFj0=OqFfea0dXdd9vqai=hGuQ8kuc9pgc9s8qqaq=dirpe0xb9q8qiLsFr0=vr0=vr0dc8meaabaqaciaacaGaaeqabaqabeGadaaakeaacqWGqbaucqGH9aqpcqaIXaqmcqGHsisldaaeWbqaamaabmaabaqbaeqabiqaaaqaaiabd6gaUbqaaiabdUgaRbaaaiaawIcacaGLPaaaaSqaaiabdUgaRjabg2da9iabicdaWaqaaiabd6eaobqdcqGHris5aOGaemiCaa3aaWbaaSqabeaacqWGRbWAaaGccqGGOaakcqaIXaqmcqGHsislcqWGWbaCcqGGPaqkdaahaaWcbeqaaiabd6gaUjabgkHiTiabdUgaRbaaaaa@47AB@

where *n *denotes the total number of the predicted domain interactions and *N *denotes the overlap of the predicted domain interactions with the gold standard set. *p *represents the probability that a randomly predicted domain pair is in the gold standard set. This measure characterizes the significance of an overlap.

## Abbreviations

PPI: Protein-Protein Interaction;

DDI: Domain-Domain Interaction;

LPM: Linear Programming with Multi-domain pairs;

APMM: Association Probabilistic Method with Multi-domain pairs;

EM: Expectation Maximization;

LP: Linear Programming;

ASNM: Association Numerical Method;

RMSE: Root Mean Square Error;

IST: Interaction Sequence Tags;

AUC: Area Under Curve.

## Authors' contributions

RSW and YW designed and implemented the algorithm. LYW and XSZ gave some helpful suggestions on the experiments. LC proposed the main idea and gave many helpful suggestions for the manuscript. All authors wrote and approved the manuscript.

## Supplementary Material

Additional file 1 This additional file contains the detected superdomains and cooperative domains from DIP (Tables I-III), cooperative-domain interactions verified by PDB crystal structure (Figure I), RMSE comparison results on all protein pairs (Table IV, Table V), and the additional information on the LP model. Click here for file

## References

[B1] Eisbacher M, Holmes M, Newton A, Hogg P, Khachigian L, Crossley M, Chong B (2003). Protein-protein interaction between Fli-1 and GATA-1 mediates synergistic expression of megakaryocyte-specific genes through cooperative DNA binding. Mol Cell Biol.

[B2] Labalette C, Renard C, Neuveut C, Buendia M, Wei Y (2004). Interaction and functional cooperation between the LIM protein FHL2, CBP/p300, and β-Catenin. Mol Cell Biol.

[B3] Salwinski L, Miller C, Smith A, Pettit F, Bowie J, Eisenberg D (2004). The database of interacting proteins: 2004 update. Nucl Acids Res.

[B4] Mewes H, Frishman D, Mayer K, Munsterkotter M, Noubibou O, Pagel P, Rattei T, Oesterheld M, Ruepp A, Stumpflen V (2006). MIPS: analysis and annotation of proteins from whole genomes in 2005. Nucl Acids Res.

[B5] Deng M, Sun F, Chen T (2003). Assessment of the reliability of protein-protein interactions and protein function prediction. Pac Symp Biocomput.

[B6] von Mering C, Krause R, Snel B, Cornell M, Oliver S, Fields S, Bork P (2002). Comparative assessment of large-scale data sets of prote-protein interactions. Nature.

[B7] Enright AJ, Iliopoulos KNOC I (1999). Protein interaction maps for complete genomes based on gene fusion events. Nature.

[B8] Marcotte E, Pellegrini M, Ng H, Rice D, Yeates T, Eisenberg D (1999). Detecting protein function and protein-protein interactions from genome sequences. Science.

[B9] Pellegrini M, Marcotte E, Thompson M, Eisenberg D, Yeates T (1999). Assigning protein functions by comparative genome analysis: Protein phylogenetic profiles. Proc Natl Acad Sci.

[B10] Szilagyi A, Grimm V, Arakaki A, Skolnick J (2005). Prediction of physical protein-protein interactions. Phys Biol.

[B11] Sprinzak E, Margalit H (2001). Correlated sequence-signatures as markers of protein-protein interaction. J Mol Biol.

[B12] Deng M, Mehta S, Sun F, Chen T (2002). Inferring domain-domain interactions from protein-protein interactions. Genome Res.

[B13] Chen L, Wu L, Y W, Zhang X (2006). Inferring protein interactions from experimental data by association probabilistic method. Proteins.

[B14] Liu Y, Liu N, Zhao H (2005). Inferring protein-protein interactions through high-throughput interaction data from diverse organisms. Bioinformatics.

[B15] Dohkan S, Koike A, Takagi T (2003). Support vector machines for predicting protein-protein interactions. Genome Inform.

[B16] Hayashida M, Ueda N, Akutsu T (2003). Inferring strengths of protein protein interactions from experimental data using linear programming. Bioinformatics.

[B17] Riley R, Lee C, Sabatti C, Eisenberg D (2005). Inferring protein domain interactions from databases of interacting proteins. Genome Biol.

[B18] Lee H, Deng M, Sun F, Chen T (2006). An integrated approach to the prediction of domain-domain interactions. BMC Bioinformatics.

[B19] Guimaraes K, Jothi R, Zotenko E, Przytycka T (2006). Predicting domain-domain interactions using a parsimony approach. Genome Biol.

[B20] Moza B, Buonpane R, Zhu P, Herfst C, Rahman A, McCormick J, Kranz D, Sundberg E (2006). Long-range cooperative binding effects in a T cell receptor variable domain. Proc Natl Acad Sci.

[B21] Bateman A, Coin L, Durbin R, Finn R, Hollich V, Griffiths-Jones S, Khanna A, Marshall M, Moxon E, Sonnhammer S, Studholme D, Yeats C, Eddy S (2004). The Pfam protein families database. Nucl Acids Res.

[B22] Han D, Kim H, Seo J, Jang W (2003). A domain combination based probabilistic framework for protein-protein interaction prediction. Genome Inform.

[B23] Han D, Kim H, Jang W, Lee S, Suh J (2004). PreSPI: a domain combination based prediction system for protein-protein interaction. Nucl Acids Res.

[B24] Wang M, Caetano-Anolles G (2006). Global phylogeny determined by the combination of protein domains in proteomes. Mol Biol Evol.

[B25] Klemm J, Pabo C (1996). Oct-1 POU domain-DNA interactions: cooperative binding of isolated subdomains and effects of covalent linkage. Genes Dev.

[B26] Via A, Zanzoni A, Helmer-Citterich M (2005). Seq2Struct: a resource for establishing sequence-structure links. Bioinformatics.

[B27] Kundrotas P, Alexov E (2006). PROTCOM: searchable database of protein complexes enhanced with domain-domain structures. Nucl Acids Res.

[B28] Ito T, Chiba T, Ozawa R, Yoshida M, Hattori M, Sakaki Y (2001). A comprehensive two hybrid analysis to explore the yeast protein interactome. Proc Natl Acad Sci.

[B29] Krogan N, Cagney G, Yu H, Zhong G, Guo X, Ignatchenko A, Li J, Pu S, Datta N, Tikuisis A, Punna T, Peregrin-Alvarez J, Shales M, Zhang X, Davey M, Robinson M, Paccanaro A, Bray J, Sheung A, Beattie B, Richards D, Canadien V, Lalev A, Mena F, Wong P, Starostine A, Canete M, Vlasblom J, Wu S, Orsi C, Collins S, Chandran S, Haw R, Rilstone J, Gandi K, Thompson N, Musso G, St Onge P, Ghanny S, Lam M, Butland G, Altaf-Ul A, Kanaya S, Shilatifard A, O'Shea E, Weissman J, Ingles C, Hughes T, Parkinson J, Gerstein M, Wodak S, Emili A, Greenblatt J (2006). Global landscape of protein complexes in the yeast Saccharomyces cerevisiae. Nature.

[B30] Finn R, Marshall M, Bateman A (2005). iPfam: visualization of protein-protein interactions in PDB at domain and amino acid resolutions. Bioinformatics.

[B31] Ng S, Zhang Z, Tan S, Lin K (2003). InterDom: a database of putative interacting protein domains for validating predicted protein interactions and complexes. Nucl Acids Res.

[B32] PreSPI. http://prespi.icu.ac.kr.

[B33] Apic G, Gough J, Teichmann S (2001). An insight into domain combinations. Bioinformatics.

[B34] Shevchenko A, Schaft D, Roguev A, Pijnappel WWMP, Stewart A, Shevchenko A (2002). Deciphering protein complexes and protein interaction networks by tandem affinity purification and mass spectrometry. Mol Cell Proteomics.

[B35] Gavin A, Aloy P, Grandi P, Krause R, Boesche M, Marzioch M, Rau C, Jensen L, Bastuck S, Dümpelfeld B, Edelmann A, Heurtier M, Hoffman V, Hoefert C, Klein K, Hudak M, Michon A, Schelder M, Schirle M, Remor M, Rudi T, Hooper S, Bauer A, Bouwmeester T, Casari G, Drewes G, Neubauer G, Rick J, Kuster B, Bork P, Russell R, Superti-Furga G (2006). Proteome survey reveals modularity of the yeast cell machinery. Nature.

[B36] Aloy P, Russell R (2006). Structural systems biology: modelling protein interactions. Nat Rev Mol Cell Biol.

[B37] Aloy P, Bottcher B, Ceulemans H, Leutwein C, Mellwig C, Fischer S, Gavin A, Bork P, Superti-Furga G, Serrano L, Russell R (2004). Structure-based assembly of protein complexes in yeast. Science.

[B38] Schneidman-Duhovny D, Inbar Y, Nussinov R, Wolfson H (2005). PatchDock and symmDock: servers for rigid and symmetric docking. Nucl Acids Res.

[B39] Letunic I, Copley R, Schmidt S, Ciccarelli F, Doerks T, Schultz J, Ponting C, Bork P (2004). SMART 4.0: towards genomic data integration. Nucl Acids Res.

[B40] Gene Ontology Database. http://www.geneontology.org.

[B41] Chen Y, Xu D (2004). Global protein function annotation through mining genome-scale data in yeast Saccharomyces cerevisiae. Nucl Acids Res.

[B42] Wu H, Su Z, Mao F, Olman V, Xu Y (2005). Prediction of functional modules based on comparative genome analysis and Gene Ontology application. Nucl Acids Res.

