# Supplementary material for: Analysis on multi-domain cooperation for predicting protein-protein interactions
Source: BMC Bioinformatics. 2007 Oct 16;8:391. doi: 10.1186/1471-2105-8-391 (PMC2222654; doi:10.1186/1471-2105-8-391)
Supplement: Additional file 1 — This additional file contains the detected superdomains and cooperative domains from DIP (Tables I-III), cooperative-domain interactions verified by PDB crystal structure (Figure I), RMSE comparison results on all protein pairs (Table IV, Table V), and the additional information on the LP model. [file 1471-2105-8-391-S1.pdf]

Additional file

A Detected superdomains and cooperative domains in DIP data by APMM

Table I: Superdomains by our method from DIP protein interaction data, where GO annotations are denoted in italic.

Superdomains	Descriptions	GO similarity
PF08022, PF01794	(1) FAD-binding domain<br>(2) Ferric reductase like transmembrane component, *integral to membrane, Function iron ion binding, FAD binding, oxidoreductase activity, Process electron transport*	
PF07687, PF01546	(1) Peptidase dimerisation domain, *hydrolase activity, protein dimerization activity*<br>(2) Peptidase family M20/M25/M40, *metallopeptidase activity, proteolysis*	1-1-3-16<br>1-1-3-16-18-6<br>Similarity: 4
PF07717, PF04408	(1) Domain of unknown function (DUF1605), *ATP binding, ATP-dependent helicase activity*<br>(2) Helicase associated domain (HA2), *helicase activity*	1-1-3-14-1<br>1-1-3-14<br>Similarity: 4
PF04810, PF04815	(1) Sec23/Sec24 zinc finger, *COPII vesicle coat, protein binding, zinc ion binding, intracellular protein transport, ER to Golgi vesicle-mediated transport*<br>(2) Sec23/Sec24 helical domain, *COPII vesicle coat, protein binding, intracellular protein transport, ER to Golgi vesicle-mediated transport*	3-1-1-1-24-1-10-1-28-1-3-2-3-1<br>3-1-1-1-24-1-10-1-28-1-3-2-3-1<br>Similarity: 14
PF02866, PF00056	(1) lactate/malate dehydrogenase, alpha/beta C-terminal domain, *oxidoreductase activity, tricarboxylic acid cycle intermediate metabolism*<br>(2) lactate/malate dehydrogenase, NAD binding domain, *oxidoreductase activity, tricarboxylic acid cycle intermediate metabolism*	2-1-2-5-11-17-2-4-7<br>2-1-2-5-11-17-2-4-7<br>Similarity: 9
PF00562, PF04567	(1) RNA polymerase Rpb2, domain 6, *DNA-directed RNA polymerase activity, DNA binding, transcription*<br>(2) RNA polymerase Rpb2, domain 5, *DNA-directed RNA polymerase activity, DNA binding, transcription*	1-1-3-39-16-3-16<br>1-1-3-39-16-3-16<br>Similarity: 7
PF00113, PF03952	(1) Enolase, C-terminal TIM barrel domain, *phosphopyruvate hydratase complex, phosphopyruvate hydratase activity, glycolysis*<br>(2) Enolase, N-terminal domain, *phosphopyruvate hydratase complex, phosphopyruvate hydratase activity, glycolysis*	3-1-1-1-24-1-10-1-30-1-28<br>3-1-1-1-24-1-10-1-30-1-28<br>Similarity: 12
PF00408, PF02879	(1) Phosphoglucomutase/phosphomannomutase, C-terminal domain, *intramolecular transferase activity, phosphotransferases, carbohydrate metabolism*<br>(2) Phosphoglucomutase/phosphomannomutase, alpha/beta/alpha domain II, *intramolecular transferase activity, phosphotransferases, carbohydrate metabolism*	1-1-3-18-7-6<br>1-1-3-18-7-6<br>Similarity: 6

Table II: Putative cooperative domains by our method from DIP protein interaction data, where GO annotations are denoted in italic.

Interactor(s)I	Description	Interactor II	Description
PF00097, PF00176	(1) Zinc finger, C3HC4 type (RING finger), *protein binding, zinc ion binding* (2) SNF2 family N-terminal domain, *DNA binding, ATP binding*	PF00125	Core histone H2A/H2B/H3/H4, *DNA binding*
PF00806, PF00076	(1) Pumilio-family RNA binding repeat, *RNA binding* (2) RNA recognition motif. (a.k.a. RRM, RBD, or RNP domain), nucleic acid binding	PF00660	Seripauperin and TIP1 family, response to stress
PF08389, PF03810	(1) Exportin 1-like protein (2) Importin-beta N-terminal domain, *nuclear pore, nucleus, cytoplasm, protein transporter activity, protein import into nucleus, docking*	PF00638	RanBP1 domain, intracellular transport
PF00515, PF00160	(1) Tetratricopeptide repeat (2) Cyclophilin type peptidyl-prolyl cis-trans isomerase/CLD, *protein folding*	PF00183	Hsp90 protein, unfolded protein binding, protein folding
PF00400 PF00646	(1) WD domain, G-beta repeat (2) F-box domain	PF00888	Cullin family, *cell cycle*
PF00439, PF00271	(1) Bromodomain, involved in protein-protein interactions (2) Helicase conserved C-terminal domain, *ATP binding, helicase activity, nucleic acid binding*	PF04433	SWIRM domain, mediate protein-protein interactions in the assembly of chromatin-protein complexes
PF02985, PF03810	(1) HEAT repeat (2)Importin-beta N-terminal domain, *nuclear pore, nucleus, cytoplasm, protein transporter activity, protein import into nucleus, docking*	PF04096	Nucleoporin autopeptidase, *nuclear pore, transport*
PF00023, PF02292	(1) Ankyrin repeat, one of the most common protein-protein interaction motifs (2) APSES domain, *DNA binding, transcription factor activity, regulation of transcription, DNA-dependent*	PF00498	FHA domain
PF00627, PF00240	(1) UBA/TS-N domain (2) Ubiquitin family, *protein modification*	PF01399	PCI domain
PF00560, PF00646	(1) Leucine Rich Repeat, *transferase activity* (2)F-box domain	PF00241	Cofilin/tropomyosin-type actin-binding protein, *intracellular, actin binding*

Table III: Putative strongly cooperative domains by our method from DIP protein interaction data, where GO annotations are denoted in italic.

Interactor(s)I	Description	Interactor II	Description
PF05000, PF04997	(1) RNA polymerase Rpb1, domain 4, *DNA-directed RNA polymerase activity, DNA binding, transcription*<br>(2) RNA polymerase Rpb1, domain 1, *DNA-directed RNA polymerase activity, DNA binding, transcription*	PF01193	RNA polymerase Rpb3/Rpb11 dimerisation domain, *DNA-directed RNA polymerase activity, protein dimerization activity, transcription*
PF02867, PF00317	(1) Ribonucleotide reductase, barrel domain, *ribonucleoside-diphosphate reductase complex, ribonucleoside-diphosphate reductase activity, DNA replication*<br>(2) Ribonucleotide reductase, all-alpha domain	PF03477	ATP cone domain
PF00562 PF04561	(1) RNA polymerase Rpb2, domain 6, *DNA-directed RNA polymerase activity, DNA binding, transcription*<br>(2)RNA polymerase Rpb2, domain 2, *DNA-directed RNA polymerase activity, DNA binding, transcription*	PF01193	RNA polymerase Rpb3/Rpb11 dimerisation domain, *DNA-directed RNA polymerase activity, protein dimerization activity, transcription*
PF01237, PF00023	(1) Oxysterol-binding protein, *steroid metabolism* (2) Ankyrin repeat	PF00635	MSP (Major sperm protein) domain, *structural molecule activity*
PF04408, PF00575	(1) Helicase associated domain (HA2), *helicase activity*<br>(2) S1 RNA binding domain, *RNA binding*	PF00098	Zinc knuckle, *zinc ion binding, nucleic acid binding*
PF0051, PF06424	(1) Tetratricopeptide repeat<br>(2) PRP1 splicing factor, N-terminal, *nucleus, nuclear mRNA splicing, via spliceosome*	PF01423	LSM domain, *ribonucleoprotein complex, mRNA metabolism*
PF00627, PF01412	(1) UBA/TS-N domain<br>(2)Putative GTPase activating protein for Arf, *regulation of GTPase activity*	PF08226	Domain of unknown function (DUF1720)
PF00514, PF01749	(1) Armadillo/beta-catenin-like repeat<br>(2) Importin beta binding domain, *protein transporter activity, intracellular protein transport, protein import into nucleus*	PF00583	Acetyltransferase (GNAT) family, *N-acetyltransferase activity*
PF00250, PF00498	(1) Fork head domain, *nucleus, sequence-specific DNA binding, transcription factor activity, regulation of transcription, DNA-dependent*<br>(2)FHA domain	PF01008	Initiation factor 2 subunit family, cellular biosynthesis
PF00806, PF00076	(1) Pumilio-family RNA binding repeat, *RNA binding*<br>(2) RNA recognition motif, *nucleic acid binding*	PF03381	LEM3 (ligand-effect modulator 3) family / CDC50 family, *membrane, molecular function unknown*

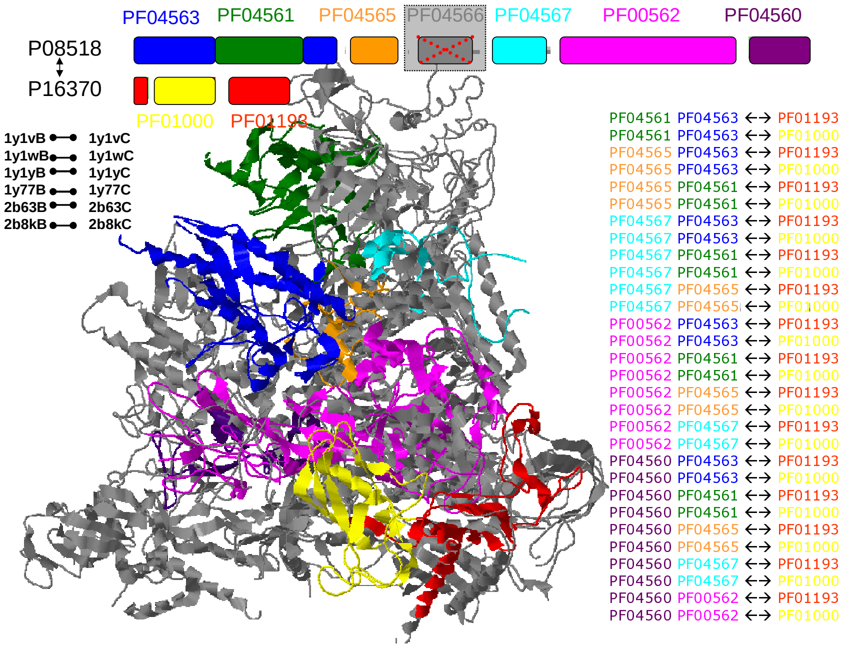

Figure I: Cooperative domain interactions for proteins P08518 (YOR151C) and P16370 (YIL021W) in DNA-directed RNA polymerase complex. Protein sequences are shown using thick gray lines, and Pfam domain annotations are shown using colored rectangular boxes (the gray ones are not shown in cooperative domains) and drawn to scale (based on the Pfam database). The names of the protein sequences in this protein complex are listed to the left of the domain architecture. The identified cooperative domain pairs are list below the domain architecture. The domain names are labeled by the same color as in the Pfam domain annotation. The cartoon of PDB crystal structure (PDB ID: 1y1v, DNA-directed RNA polymerase) demonstrates cooperative-domain interactions with domain colors consistent with the domain annotation. Other complexes in PDB containing the founded cooperative domains are list by their matched PDB IDs and chain IDs.

B Cooperative-domain interactions for proteins P08518 (YOR151C) and P16370 (YIL021W) in DNA-directed RNA polymerase complex

Cooperative domain interactions for proteins P08518 (YOR151C) and P16370 (YIL021W) in DNA-directed RNA polymerase complex are shown in Figure I.

Table IV: Comparisons of RMSE and training time for Ito's Yeast Interaction Datasets (YIP)

	EM	ASSOC	ASNM	**LPM**	**APMM**
Train					
1st	0.4835	0.4601	0.0367	0.0142	0.0115
2nd	0.4829	0.4564	0.0415	0.0150	0.0127
3rd	0.4745	0.4472	0.0413	0.0120	0.0102
4th	0.4824	0.4594	0.0402	0.0124	0.0107
5th	0.4909	0.4598	0.0397	0.0150	0.0123
Average	0.4828	0.4566	0.0399	0.0137	0.0115
Time(seconds)	0.8254	0.0060	0.0058	1.093	0.0928
Test					
1st	0.6281	0.6121	0.0771	0.0425	0.0444
2nd	0.6075	0.5747	0.0458	0.0293	0.0307
3rd	0.6394	0.5687	0.0719	0.0475	0.0467
4th	0.5906	0.5606	0.0702	0.0462	0.0456
5th	0.6628	0.5960	0.0660	0.0405	0.0507
Average	0.6257	0.5824	0.0662	0.0412	0.0436

Table V: Comparisons of RMSE and training time for Krogan's Yeast Extended Datasets

	EM	ASSOC	ASNM	**LPM**	**APMM**
Train					
1st	0.4136	0.4473	0.4382	0.1285	0.1370
2nd	0.4151	0.4461	0.4324	0.1297	0.1339
3rd	0.4152	0.4447	0.4345	0.1281	0.1336
4th	0.4159	0.4456	0.4349	0.1296	0.1356
5th	0.4159	0.4462	0.4351	0.1313	0.1353
Average	0.4151	0.4460	0.4350	0.1294	0.1351
Time(seconds)	2814.2	0.2034	0.203	118.66	6.4718
Test					
1st	0.5338	0.5309	0.4609	0.4027	0.3505
2nd	0.5483	0.5431	0.4853	0.3726	0.3411
3rd	0.5465	0.5418	0.4740	0.3938	0.3498
4th	0.5376	0.5409	0.4760	0.3577	0.3253
5th	0.5435	0.5446	0.4886	0.3652	0.3357
Average	0.5419	0.5403	0.4769	0.3784	0.3405

C RMSE on all protein pairs of Ito's YIP

In order to demonstrate the effect of cooperative domain interactions on the improvement of accuracy, we compute RMSE only on those protein pairs containing cooperative-domain pairs for cross-validation. RMSE comparison results on all protein pairs are given in Tables IV and V, which clearly show that the proposed methods, LPM and APMM with the consideration of multi-domain pairs outperform other methods.

Here we give an example to illustrate why the model with multiple-domain pairs can improve the accuracy of predictions. In Ito's data, (P23561,P25344) is a pair of proteins with interaction probability 0.156250. P23561 has two domains: IPR001660 and CLUS00003. P25344 has one domain: IPR000159. If we only consider two-domain pairs, there are two pairs of domains in this protein pair, i.e., (IPR001660, IPR000159), and (CLUS00003, IPR000159). According to APM, the interaction probabilities of these two pairs of domains are respectively 0.081441 and 0.043325. Then according to the prediction formula Eq.(1), the interaction probability of (P23561,P25344) is 0.121238 which has a big difference with the original observed probability 0.156250. If we consider multiple-domain pairs (including two-domain pairs), there are three pairs of domains in these two proteins: (IPR001660, IPR000159), (CLUS00003, IPR000159) and (IPR001660, CLUS00003; IPR000159), among which only ({IPR001660, CLUS00003}, IPR000159) is not deleted based on the variable deleting strategy. In other words, its probability is the highest among those three pairs of domains. Specifically, its interaction probability is 0.156250 which is computed by APMM. Hence, (P23561,P25344)

is predicted as an interaction protein pair with probability 0.156250 which is consistent with the observed probability.

D Inference models for domain interaction

In the following, we first change the mathematical programming model in the main text into a standard LP form. Letting $\varepsilon_{ij}^k = u_{ij}^k - v_{ij}^k$ and $u_{ij}^k \geq 0, v_{ij}^k \geq 0$, then the mathematical programming model can be transformed into the following form:

$$\begin{aligned}
\min_{u_{ij}^k, v_{ij}^k; x_{m,n}; x_{mr,n}; x_{m,nr}} \quad & \sum_{P_{ij}^k} (u_{ij}^k + v_{ij}^k) \\
s.t. \quad & \sum_{D_{m,n} \in P_{ij}^k} x_{m,n} + \sum_{D_{mr,n} \in P_{ij}^k} x_{mr,n} + \sum_{D_{m,nr} \in P_{ij}^k} x_{m,nr} = \beta_{ij}^k - u_{ij}^k + v_{ij}^k \\
& x_{m,n} \leq 0, x_{mr,n} \leq 0, x_{m,nr} \leq 0 \\
& u_{ij}^k \geq 0, v_{ij}^k \geq 0 \\
& i, j \in 1, \ldots, N_k; k = 1, \cdots, K
\end{aligned} \tag{I}$$

Due to the additional variables (the variables that three-domain pairs correspond to) introduced in (I), there may exist multiple optimal solutions. Next, we further add a term in the objective function so as to find a solution with a sparse structure on the protein interaction network.

Actually, sparse principle is widely adopted in a variety of reconstruction problems of biological networks owing to the evolutional implication. The sparse principle in protein interaction network means that we use as few as possible interacting domain pairs to explain the observed protein-protein interactions, which is also considered to be biologically plausible. It is based on the consideration that in a pair of interacting proteins, there are only a few interacting domain pairs among all domain pairs associated with this protein pair, and a pair of domains which appears in many interacting protein pairs is more likely to interact with each other. Also many research works indicate that the protein interaction network is sparse in nature. Thus we assume sparseness is one of its basic topological properties of domain interaction network and can incorporate this information in the inference of domain interactions. Therefore, by adding a term to drive the inferred domain interaction network to be sparse, another linear programming model can be obtained.

$$\begin{aligned}
\min_{u_{ij}^k; v_{ij}^k; x_{m,n}; x_{mr,n}; x_{m,nr}} \quad & \sum_{P_{ij}^k} (u_{ij} + v_{ij}) - \lambda \sum (x_{m,n} + x_{mr,n} + x_{m,nr}) \\
s.t. \quad & \sum_{D_{m,n} \in P_{ij}^k} x_{m,n} + \sum_{D_{mr,n} \in P_{ij}^k} x_{mr,n} + \sum_{D_{m,nr} \in P_{ij}^k} x_{m,nr} = \beta_{ij}^k - u_{ij}^k + v_{ij}^k \\
& x_{m,n} \leq 0, x_{mr,n} \leq 0, x_{m,nr} \leq 0 \\
& u_{ij}^k \geq 0, v_{ij}^k \geq 0 \\
& i, j \in 1, \ldots, N_k; k = 1, \cdots, K
\end{aligned} \tag{II}$$

where λ is introduced as a positive parameter that balances the error and sparsity terms in the objective function. In other words, the second term of the objective function is to force the solution as sparse as possible. Hence, by solving (II), we can obtain $x_{m,n}, x_{mr,n}$ and $x_{m,nr}$. Then the domain interactions can be straightforwardly calculated by $\Pr(d_{m,n} = 1) = 1 - e^{x_{m,n}}$, $\Pr(d_{mr,n} = 1) = 1 - e^{x_{mr,n}}$ and $\Pr(d_{m,nr} = 1) = 1 - e^{x_{m,nr}}$.
